# Janus-type emission from a cyclometalated iron(iii) complex

**DOI:** 10.1038/s41557-023-01137-w

**Published:** 2023-02-27

**Authors:** Jakob Steube, Ayla Kruse, Olga S. Bokareva, Thomas Reuter, Serhiy Demeshko, Roland Schoch, Miguel A. Argüello Cordero, Athul Krishna, Stephan Hohloch, Franc Meyer, Katja Heinze, Oliver Kühn, Stefan Lochbrunner, Matthias Bauer

**Affiliations:** 1 Institute of Inorganic Chemistry, Paderborn University, Paderborn, Germany; 2 Center for Sustainable Systems Design, Paderborn University, Paderborn, Germany; 3 Department of Life, Light and Matter, University of Rostock, Rostock, Germany; 4 Institute for Physics, University of Rostock, Rostock, Germany; 5 Institute of Physics, University of Kassel, Kassel, Germany; 6 Department of Chemistry, Johannes Gutenberg University, Mainz, Germany; 7 Institute of Inorganic Chemistry, University of Göttingen, Göttingen, Germany; 8 Institute of General, Inorganic and Theoretical Chemistry, University of Innsbruck, Innsbruck, Austria

**Keywords:** Light harvesting, Photocatalysis, Excited states, Density functional theory, Sustainability

## Abstract

Although iron is a dream candidate to substitute noble metals in photoactive complexes, realization of emissive and photoactive iron compounds is demanding due to the fast deactivation of their charge-transfer states. Emissive iron compounds are scarce and dual emission has not been observed before. Here we report the Fe^III^ complex [Fe(ImP)_2_][PF_6_] (HImP = 1,1′-(1,3-phenylene)bis(3-methyl-1-imidazol-2-ylidene)), showing a Janus-type dual emission from ligand-to-metal charge transfer (LMCT)- and metal-to-ligand charge transfer (MLCT)-dominated states. This behaviour is achieved by a ligand design that combines four *N*-heterocyclic carbenes with two cyclometalating aryl units. The low-lying π* levels of the cyclometalating units lead to energetically accessible MLCT states that cannot evolve into LMCT states. With a lifetime of 4.6 ns, the strongly reducing and oxidizing MLCT-dominated state can initiate electron transfer reactions, which could constitute a basis for future applications of iron in photoredox catalysis.

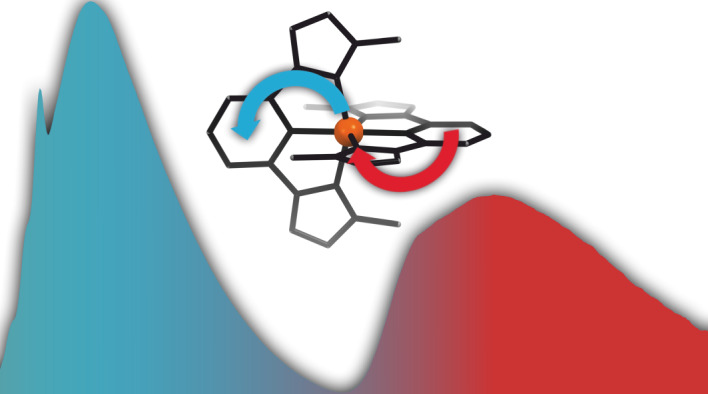

## Abstract (web summary)

Noble metals dominate the field of photosensitizers and luminophores. Now, an approach incorporating cyclometalating and carbene functions into Fe^III^ complexes has been shown to enable dual emission from the opposing ligand-to-metal and metal-to-ligand charge-transfer states. The latter shows an exceptionally long lifetime of 4.6 ns and is quenched by oxygen and other quenchers.

## Main

The greatest challenge in the search for photoactive iron complexes is posed by the weak ligand field splitting of *t*_2g_- and *e*_g_*-based orbitals, which is smaller in 3*d* transition metal complexes compared with their 4*d* and 5*d* counterparts^1^. The low-energy metal-centred states act as dark excited-state traps, quenching potentially emissive metal-to-ligand charge-transfer (MLCT) or ligand-to-metal charge-transfer (LMCT) states. Consequently, short lifetimes are observed for charge-transfer states^2^. Attempts to invert the order of metal-centred and charge-transfer states focus on the destabilization of metal-centred levels by strong *σ* donors^3–5^ or the stabilization of charge-transfer states by *π* acceptors^6–9^. In Fe^II^ complexes, these strategies typically lead to MLCT lifetimes in the picosecond range. Exceptionally long MLCT lifetimes could be obtained: (1) with six *N*-heterocyclic carbene (NHC) donor groups (*τ* = 0.5 ns)^10^; and (2) using the HOMO inversion concept^11^ (*τ* = 2.7 ns)^12^. Only recently, one emissive mononuclear Fe^II^ complex was reported^13,14^, while two emissive Fe^III^ complexes possessing six electron-donating carbene donor units have been reported so far^15,16^. The Fe^III^ congener of the Fe^II^ complex with an MLCT lifetime of 0.5 ns presented by Wärnmark et al.^15^ exhibits fluorescence from a ^2^LMCT state with a lifetime of ~100 ps. Rigid anionic tripodal carbenes achieve a ^2^LMCT lifetime of 2 ns, with a quantum yield of 2% in aerated MeCN solution^16^. Due to the electron-rich nature of NHC ligands and the electron-poor nature of the *d* ^5^ electron configuration, MLCT emission could not yet be observed in emissive Fe^III^ complexes. Cyclometalating phenyl-containing ligands offer strong *σ*-donor but also *π*-donor properties. More importantly, they show lower-energy and thus more easily accessible π* orbitals than NHC donors. Fe^II^ complexes with such ligand types were extensively studied in theoretical investigations by Jakubikova and Dixon^17–20^. These predictions were recently supported by experimental studies^14,21^. The Fe^II^ complex, derived from [Fe(tpy)_2_]^2+^ (tpy = 2,2′:6′,2′′-terpyridine) by exchanging one tpy with a deprotonated phenylbipyridine, showed an extension of the MLCT lifetime by a factor of five and a decrease of the metal-centred state lifetime, while cyclometalating phenylphenanthroline ligands deliver a luminescent Fe^II^ complex with a ^3^MLCT lifetime of ~1 ns^14^. Here we demonstrate that cyclometalating ligands in combination with NHC donors coordinated to Fe^III^ enable dual emission—namely from ^2^LMCT excited states and additionally from ^2^MLCT excited states at higher energy, enabled by stabilization of the Fe^IV^ oxidation state and energetically accessible π* orbitals, due to the unique ligand design.

## Results and discussion

### Ground-state characterization

**Fig. 1:**
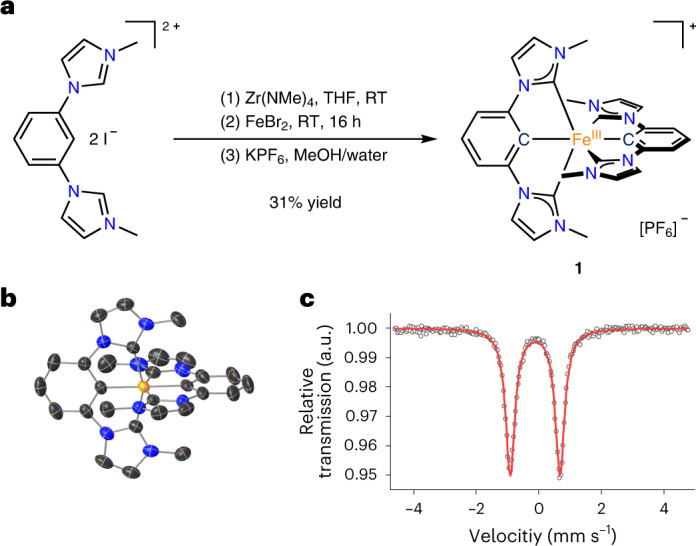
Synthesis, X-ray structure and Mössbauer spectrum of 1. **a**, Activation of the pro-ligand using a zirconium reagent with subsequent transmetalation onto iron. MeOH, methanol; RT, room temperature; THF, tetrahydrofuran. Due to the donor strength of the ligand, the Fe^II^ complex is oxidized under air to the Fe^III^ complex **1**. **b**, Structure of the cation of **1**, as determined by X-ray diffraction. Hydrogen atoms and counter ion are omitted for clarity. **c**, Mössbauer spectrum of **1** at 80 K showing the characteristic doublet of a low-spin Fe^III^ complex. Source data

The complex [Fe(ImP)_2_][PF_6_] (HImP = 1,1′-(1,3-phenylene)bis(3-methyl-1-imidazol-2-ylidene)) **1** is obtained by transmetalation of the ligand ImP^−^ (Fig. 1a)^22–24^, resulting in a blue air- and water-stable Fe^III^ complex as [PF_6_]^−^ salt after workup under atmospheric air. Chemical integrity and purity were confirmed by NMR spectroscopy (Supplementary Fig. 1), mass spectrometry (Supplementary Fig. 2) and elemental analysis.

In the crystal structure (Fig. 1b), the C_NHC_–Fe–C_NHC_ bite angle of 155° is smaller than in terpyridine analogues^4^ and leads to a more distorted octahedral geometry. The ligand-to-ligand dihedral angle is 86°. The doublet found in the Mössbauer spectrum of Fig. 1c with an isomer shift of −0.12 mm s^−1^ and a quadrupole splitting of 1.59 mm s^−1^ indicates an *S* = ½ configuration, corresponding to a *d* ^5^ low-spin complex. Low isomer shifts are common for iron complexes with multiple NHC donors^15,16^ and the very low value for **1** can be explained by the short Fe–C bonds (1.98 Å for Fe–C_NHC_ and 1.94 Å for Fe–C_CM_), which lead to a compressed 4*s* orbital and hence a higher 4*s* electron density at the iron nucleus^25^. Temperature-dependent magnetic susceptibility measurements (Supplementary Fig. 6) show the typical behaviour of a low-spin Fe^III^ compound with molar magnetic susceptibility and temperature product *χ*_M_ *T* values higher than the spin-only value (0.375 cm^3^ mol^–1^ K versus 0.49–0.64 cm^3^ mol^–1^ K in **1**) and the expected deviation from the Curie law due to non-quenched orbital momentum of the ^2^ *T*_2_ ground state.

**Fig. 2:**
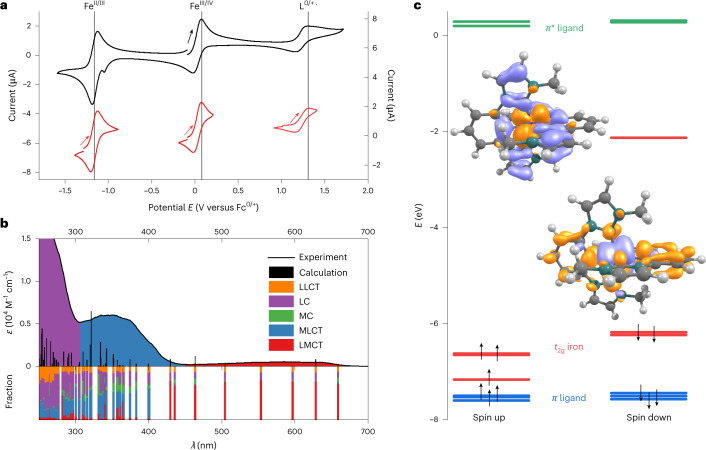
Electrochemical, optical and electronic properties of 1. **a**, Cyclic voltammogram of **1** (10^−3^ M) in MeCN with 0.1 M [*n*Bu_4_N][PF_6_] as the electrolyte at a scan rate of 100 mV s^−1^. Left y axis, whole voltammogram; right y axis, individual voltammograms. **b**, Ultraviolet–visible spectrum of **1** in MeCN (10^−4^ M) with TDDFT-calculated transitions and contributions from ligand-to-ligand charge-transfer (LLCT), ligand-centred *π*–π* (LC), metal-centred (MC), LMCT and MLCT states. **c**, Molecular orbital scheme showing the highest occupied orbitals (*t*_2g_ orbitals (red) and ligand-based orbitals (blue)) and the lowest unoccupied orbitals (π* orbitals of the ligand moiety (green)). The transition densities of the dominant LMCT (left) and MLCT (right) transitions are also depicted (hole, purple; electron, orange). Source data

The tremendous effect of cyclometalation on the electronic structure is revealed by cyclic voltammetry (Fig. 2a). A reversible Fe^II/III^ redox wave is found at −1.16 V versus FcH°^/+^. Compared with the value of 0.31 V for the analogous iron complex with two C^N^C ligands (2,6-bis(3-methyl-imidazole-1-ylidine)-pyridine), a cathodic shift of ~1.5 V is observed^26^. This corresponds to the behaviour of the [Fe(tpy)_2_]^2+^/[Fe(pbpy)(tpy)]^+^ (Hpbpy = 6-phenyl-2,2′-bipyridine) pair with a cathodic shift of ~0.8 V^21^. A quasi-reversible wave at the anodic peak potential of *E*_p,a_ = 0.08 V assigned to the Fe^III/IV^ couple is additionally detected. The irreversible wave at *E*_p,a_ = 1.31 V is attributed to ligand oxidation. An irreversible reduction of the ligand can be suspected at around −2.7 V, close to the solvent reduction (Supplementary Fig. 7).

The absorption spectrum of **1** (Fig. 2b) can be divided into three parts: the first at *λ* > 450 nm (*λ*_ε,max_ = 585 nm; *ε* = 540 M^−1^ cm^−1^; red in Fig. 2b), the second at 300 nm < *λ* < 450 nm (*λ*_ε,max_ = 351 nm; *ε* = 6,000 M^−1^ cm^−1^; blue in Fig. 2b) and the third at *λ* < 300 nm (purple in Fig. 2b). Time-dependent density functional theory (TDDFT) calculations with optimally tuned range-separated functionals suited for the description of charge-transfer states^27,28^ reveal the nature of the underlying transitions (Fig. 2b,c). Previously, it had been shown that iron complexes with NHC ligands could be reasonably described by this method unless metal-centred transitions play an important role^29^. The low-energy part of the spectrum of **1** is of charge-transfer character by design, which justifies the use of TDDFT for the assignment of the absorption spectrum (Fig. 2b,c).

The low-energy part above 450 nm is dominated by LMCT transitions caused by transitions from the ligand *π* orbital involving both imidazole and phenyl donors to the singly occupied metal-centred *d*_*π*_ acceptor orbital. The transitions contain a small amount of ligand-centred character due to the covalency of the iron–phenyl bonds. The intermediate spectral range is dominated by MLCT transitions. Contributions of ligand-centred character cannot be neglected, but predominant ligand-based transitions can be excluded since the analogous Co^III^ complex does not show transitions above 350 nm (Supplementary Fig. 8). In the MLCT band, transitions originate from singly and doubly occupied Fe^III^ *d* orbitals to the π* orbitals of the ligand, which extend over the phenyl and NHC moieties. These transitions possess some ligand-centred character, which is more pronounced than in the LMCT transitions. This is again caused by covalent iron–phenyl bonds, leading to ligand contributions in the *t*_2g_-based donor orbitals. The high-energy absorbance below 300 nm is finally dominated by ligand-centred transitions, with only minor contributions from ligand-to-ligand charge-transfer and MLCT states. For simplicity, we denote the low-, mid- and high-energy bands as LMCT, MLCT and ligand centred, respectively, and imply a mixed character with predominant contributions. The frontier orbitals are depicted in Supplementary Fig. 10 and the contributions of the metal and ligand fragments to the transitions are provided in Supplementary Fig. 12. The complementary spectra of the electrochemically generated singly reduced and oxidized species **1** ^**−**^ and **1** ^**+**^ are shown in Supplementary Fig. 13. According to DFT calculations on **1** ^**−**^ and **1** ^**+**^, these are essentially metal-centred redox processes, showing the mainly Fe^II^ and Fe^IV^ character of **1** ^**−**^ and **1** ^**+**^, respectively. These results indicate that iron-involved charge-transfer bands should be energetically lower than ligand-centred transitions. This assignment also agrees with the absence of low-energy charge-transfer transitions in the analogous Co^III^ complex (Supplementary Fig. 8).

### Excited-state characterization

**Fig. 3:**
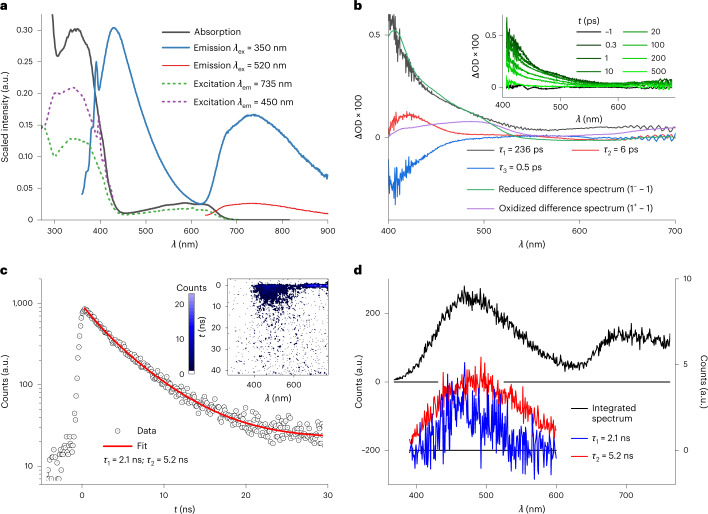
Excited-state spectroscopy and characterization of 1. **a**, Absorption and emission spectra of **1** at *λ*_ex_ = 350 and 520 nm. The excitation spectra measured at 735 and 450 nm are shown as dashed lines. **b**, DAAS with *τ*_1_ = 236 ps, *τ*_2_ = 6.1 ps and *τ*_3_ = 0.5 ps, obtained from femtosecond transient absorption data after excitation at 330 nm and compared with the reduction and oxidation difference spectra obtained from spectroelectrochemical measurements as rough models for LMCT and MLCT excited states neglecting the radical cation and radical anion character of the ligands in these excited states. Inset, transient absorption spectra at the given delay times. ΔOD, change in the optical density. **c**, Decay of the fluorescence between 390 and 600 nm obtained from SCMs (inset) of a degassed solution of **1** in MeCN after 330 nm excitation, showing a double exponential decay with time constants of 2.1 ns (27.3%) and 5.2 ns (72.7%). **d**, Time-integrated spectrum obtained from SCMs with *λ*_ex_ = 330 nm (left y axis), showing a close resemblance with the fluorescence spectrum and the amplitude spectra of the 2.1 and 5.2 ns components (right y axis). Source data

Excitation of **1** into the low-energy LMCT absorption band at 520 nm results in a broad emission mirroring the LMCT absorption band (Fig. 3a). In contrast, excitation into the high-energy absorption at 350 nm, which is dominated by MLCT contributions, results in two emission bands (Fig. 3a), leading to a visible sky-blue emission (Supplementary Fig. 14), which shows sensitivity towards oxygen (Supplementary Fig. 15). Such dual room temperature luminescence is a rare observation^30,31^, particularly for first-row transition metal complexes, and has not been observed in iron complexes. Several experimental observations provide evidence for the dual emission coming from two different emissive states in a single complex and thus violating Kasha’s rule^32^. The high-energy emission at *λ*_max_ = 430 nm and the broad band emission at *λ*_max_ = 735 nm reflect the corresponding absorption bands at 351 and 585 nm, respectively. The excitation spectra (Fig. 3a) recorded with *λ*_em_ = 450 and 735 nm match the absorption spectrum, proving that the observed dual photoluminescence indeed originates from complex **1**. The two-dimensional excitation–emission plot is shown in Supplementary Fig. 16. In agreement with the two-colour absorption composed of the two main bands at 351 and 585 nm, the high-energy emission vanishes with decreasing excitation energy.

Independent of the exact nature of the emissive states, this dual emission represents a milestone in the fields of photoactive complexes of Earth-abundant elements for which luminescence is rarely observed^33^. Following the discussion on the nature of the states contributing to the absorption bands and the shape of the emission bands, it is justified to assign the low-energy luminescence to a predominantly LMCT emissive state, while the high-energy emission originates from MLCT-dominated states for which ligand contributions cannot be fully neglected. Considering the major MLCT and LMCT contributions, the moderate Stokes shifts (80 nm/0.65 eV and 150 nm/0.43 eV, respectively, determined by the difference of the maxima in the respective broad bands) indicate ^2^MLCT and ^2^LMCT states as fluorescent states.

### Excited-state dynamics

The excited-state dynamics and excited-state landscape of complex **1** are explored by ultrafast spectroscopy. The transient absorption spectra after excitation in the MLCT-dominated band at 330 nm and the decay-associated amplitude spectra (DAAS) of a global fit are shown in Fig. 3b. The DAAS are compared with the difference spectra of the reduced **1** ^**−**^ − **1** and oxidized **1** ^**+**^ − **1** species obtained by spectroelectrochemistry as rough models for LMCT and MLCT state spectral characteristics, neglecting the radical cation and radical anion character of the ligands in these excited states, respectively (full spectra are shown in Supplementary Fig. 13)^34^. The transient spectra (Fig. 3b, inset) are dominated by a strong excited-state absorption (ESA) below 550 nm, increasing in intensity until 400 nm. Towards the red, a second, weaker ESA band follows. Three time constants are obtained from the global fit: *τ*_1_ = 236 ps ± 20 ps, *τ*_2_ = 6 ± 1 ps and *τ*_3_ = 0.5 ± 0.1 ps. The slowest component (*τ*_1_) describes the general decay of the ESA features (Fig. 3b). The strong ESA band in the blue spectral region resembles the spectrum of the Fe^II^ species **1** ^**−**^ very well while oxidation of **1** to the Fe^IV^ complex **1** ^**+**^ results only in weak absorption changes around 400 nm and is consequently not responsible for the ESA band at 400 nm. Accordingly, this time constant is assigned to the ^2^LMCT state with Fe^II^ character, which decays with a rate of 236 ps ^−1^ back to the ground state. Interestingly, in the blue spectral region of the 0.5 ps component (*τ*_3_), the DAAS is a negative mirror image of the LMCT DAAS. It therefore corresponds to a rise of the dominant blue ESA band in the DAAS of *τ*_1_ and seems to reflect a population rise in the LMCT state. The DAAS of the intermediate decay component with a time constant of 6 ps (*τ*_2_) exhibits an ESA band at 420 nm, resembling signatures of neither the Fe^II^ nor Fe^IV^ species. It might be caused by an electronic relaxation from higher-lying MLCT states. Transient absorption measurements applying pump pulses into the LMCT-dominated absorption band at 600 nm, as shown in Supplementary Fig. 18, reveal the same ESA bands observed at 330 nm excitation but a single exponential signal decay. The time constant is 240 ps, proving the assignment to a ^2^LMCT state, which is directly optically excited here.

Streak camera measurements (SCMs) with excitation at 330 nm (time resolution = 50 ps) reveal a luminescence in the spectral region above 640 nm, reflecting the ^2^LMCT emission (Fig. 3c). An additional signal in the spectral range 390–600 nm, which persists over a few nanoseconds, corresponds to the fluorescence above attributed to the ^2^MLCT states. A time constant of 220 ps is found for the ^2^LMCT emission, which is in excellent agreement with the transient absorption results. For the ^2^MLCT state, two time constants of 2.1 and 5.2 ns are obtained with fractions of 27 and 73%, respectively. The spectrally integrated signal of the ^2^MLCT emission and the corresponding fit are shown in Fig. 3d. Time-correlated single-photon counting (TCSPC) experiments support this result (Supplementary Fig. 19). However, the experimental error in the lifetime determination of the ^2^MLCT luminescence is comparably high due to the low count rate per time bin. Amplitude spectra for the two decay components were extracted from the SCM data by a global fit, as shown in Fig. 3d. The two spectra are very similar, suggesting that the two emission components result from the same state. This similarity might indicate that the decay is non-exponential, and the double exponential fit is only an approximation, resulting in an averaged lifetime of 4.6 ns. Speculatively, this behaviour can be attributed to the flexibility of the ligands, which might cause a distribution of slightly different conformations^31,35^. The ^2^MLCT decay is likely to be sensitive to conformational variations in the ensemble of complexes probed, since these variations should go along with subtle changes in the crossings between different electronic states.

**Fig. 4:**
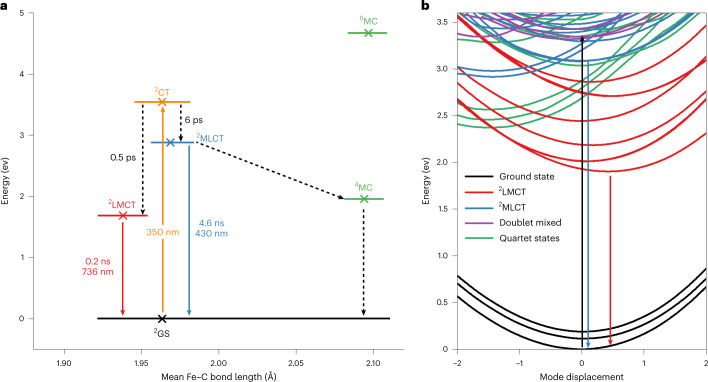
Summary of deactivation pathways and associated time scales of 1. **a**, Jablonski diagram based on the experimental results and calculated ground-state DFT energies of the optimized doublet, quartet and sextet states in their respective geometries (crosses). The ^2^LMCT and ^2^MLCT geometries are approximated from the calculated geometries of **1** ^**−**^ and **1** ^**+**^. The orange arrow indicates the excitation at 350 nm, the dotted arrows indicate non-radiative transitions, the blue arrow indicates the MLCT emission and the red arrow indicates the LMCT emission. GS, ground state; CT, charge transfer; MC, metal centred. **b**, TDDFT potential energy curves for displacement along with the a_1_ symmetry vibrational mode (in D_2d_ symmetry), showing doublet LMCT, MLCT and mixed LMCT/ligand-centred states, as well as quartet metal-centred states. The black arrow indicates the excitation at 350 nm, the blue arrow indicates the MLCT emission and the red arrow indicates the LMCT emission.

The spectroscopic results can be summarized in the following scenario of the excited-state landscape of complex **1** (Fig. 4a). Optical excitation in the near ultraviolet addresses a high density of charge-transfer states. Branching of the electronic relaxation pathways occurs shortly after the excitation. The population majority is transferred within 0.5 ps to the lowest ^2^LMCT state. This state is emissive but decays to the ground state primarily by internal conversion resulting in a lifetime of 240 ps. The weak absorption band at 600 nm (*ε* = 540 M^−1^ cm^−1^), associated with the ^2^LMCT state, shows that the corresponding transition dipole moment is small. In combination with the limited lifetime, a low quantum yield of <1% results. A minor fraction of the excited population evolves to the lowest ^2^MLCT state. The 6 ps component in the transient absorption measurements can be a signature of this relaxation. The ^2^MLCT state exhibits a lifetime of 4.6 ns and relaxes non-radiatively as well as radiatively back to the ground state, resulting in the weak but observable MLCT emission in the blue spectral region.

To rationalize the behaviour after photoexcitation, quantum chemical TDDFT calculations of potential energy curves, Huang–Rhys factors and non-adiabatic couplings were performed. The results indicate that a description of the dynamics by a simple few-state scheme, which follows from the results of TCSPC measurements and SCMs, might be an oversimplification. This consideration, together with the limited accuracy of the TDDFT method, provides a considerable bottleneck for quantitative theoretical analysis. Hence, only a qualitative picture can be drawn with the help of the potential energy curves along the symmetric mode having the strongest Huang–Rhys factor in the given energy range shown in Fig. 4b. Excitation at 350 nm targets a dense population of different doublet charge-transfer states. The many state crossings facilitate a one-electron branching into both LMCT and MLCT states. The lower-lying ^2^LMCT states are populated by a fast 0.5 ps relaxation pathway. Although quartet states (^4^MC) would, in terms of state crossings, qualify for participating in this relaxation channel, no support for the involvement of higher spin states has been found in this or other cases of luminescent *d* ^5^ systems^15,16,36^. The second pathway leading to an emissive ^2^MLCT state would require transient structural and electronic stabilization. A possible candidate for such a state has been tentatively assigned in Fig. 4. Here, already rather small energetic corrections to the potential curves beyond TDDFT could increase the barrier due to the crossing curves so as to provide a transient trapping of the population.

### Excited-state reactivity

In addition to the unique property of dual luminescence, which opens up exciting opportunities for optical applications with Earth-abundant metals, such as ratiometric O_2_ sensing^37^, the high MLCT energy and long lifetime also offer the chance for photocatalytic reactions initiated by complex **1** as a photosensitizer complementing the carbene-only ^2^LMCT sensitizer^38,39^. A first estimation of the reactivity is possible by means of excited-state potentials, which can be extracted from the electrochemical potentials and *E*_0–0_ values^40^. These were determined to be *E*_0–0,1_ = 1.9 eV for the low-energy LMCT emission and *E*_0–0,2_ = 3.1 eV for the high-energy MLCT emission. This results in excited-state redox potentials of *E* ^0^_1_(III*/II) = 0.74 V and *E* ^0^_1_(IV/III*) = −1.82 V versus FcH (1.12 V and −1.44 V versus the saturated calomel electrode) for the LMCT excited state and *E* ^0^_2_(III*/II) = 1.94 V and *E* ^0^_2_(IV/III*) = −3.02 V versus FcH (2.32 V and −2.64 V versus the saturated calomel electrode) for the MLCT excited state. According to these values, its excited LMCT state oxidation potential is similar to that of [Ru(bpy)_3_]^2+^ while its excited LMCT state reduction potential exceeds that of [Ru(bpy)_3_]^2+^ (ref. ^41^). Reduction reactions should thus be facilitated from a thermodynamic point of view. However, LMCT reactivity is kinetically disfavoured due to the sub-nanosecond lifetime. In contrast, the high-energy MLCT state combines both strongly reducing and oxidizing excited-state potentials and a nanosecond lifetime. MLCT-sensitized bimolecular reactions should thus be possible. This type of reactivity was investigated in quenching experiments using SCMs (Supplementary Fig. 20). Oxidative quenching was investigated with an excess of triethanolamine (1.0 M; *E*_ox_ = 0.46 V versus Fc)^42^, showing a reduction of the MLCT lifetime from 4.6 to 0.7 ns. An analogous result was obtained for triethylamine (2.3 M; *E*_ox_ = 0.62 V)^43^, where the MLCT lifetime was also reduced to 0.7 ns. These results agree with the excited-state potentials of 1* calculated above. Reductive quenching was investigated using benzonitrile (1.6 M; *E*_red_ = −2.76 V)^44^. Here, quenching could be observed as well, with a reduction of the MLCT lifetime to 1.0 ns. Further support for the reactivity of this state is given by the sensitization of ^1^O_2_. Although no direct observation of ^1^O_2_ emission was possible, a decrease of the fluorescence of 1,3-diphenylisobenzofuran (DPBF) under irradiation at 350 nm in the presence of **1** was observed as a probe for ^1^O_2_ generation. This corresponds to the decomposition of DPBF, which is slower than in the presence of [Ru(bpy)_3_]^2+^, but faster than without added sensitizer (Supplementary Fig. 21).

In summary, coordination of a phenylene-bis-imidazolylidene ligand to an Fe^III^ centre yields an air- and water-stable cyclometalated complex that shows two-colour luminescence from MLCT- and LMCT-dominated states. Such a unique behaviour is enabled by the ligand design incorporating strong NHC and cyclometalating *σ* and *π* donors, leading to LMCT luminescence. The phenyl/NHC π* orbitals and the stabilization of the Fe^IV^ oxidation state by NHCs/phenyl donors lead to a high-energy, yet accessible, MLCT state. Pure ligand-centred states are higher in energy. The ^2^MLCT state does not evolve into the ^2^LMCT state, probably due to the fundamental electronic differences of these states, namely Fe^IV^/radical anion versus Fe^II^/radical cation. While the ^2^LMCT state has a considerable lifetime of 240 ps, the ^2^MLCT state shows an even longer lifetime of 4.6 ns, which is the longest charge-transfer excited-state lifetime of iron complexes reported so far. Reductive and oxidative quenching experiments, as well as ^1^O_2_ sensitization, confirm an excited-state reactivity of **1**. Based on the presented data, white light emitters and multifunctional photoredox catalysts based on Earth-abundant iron might become accessible.

## Methods

### Synthesis

Dry tetrahydrofuran was obtained from an MBraun SPS-800 solvent-drying system and was subsequently stored over a 4 Å molecular sieve. ^1^H NMR spectra were recorded using Bruker Avance 500 and Bruker Ascent 700 spectrometers. Chemical shifts were calibrated to the resonance of residual undeuterated solvent. Electrospray ionization mass spectrometry spectra were recorded with a Waters Synapt G2 Quadrupole Time-of-Flight spectrometer. Iron(ii) bromide (99.8%) was purchased from Sigma–Aldrich and tetrakis(dimethylamino)zirconium was purchased from abcr. 1,1’-(1,3-phenylene)bis(3-methyl-1-imidazolium) diiodide was synthesized based on literature methods^45^.

The synthesis of bis(2,6-bis(3-methylimidazol-1-ylidene)phenyl)iron(iii) hexafluorophosphate ([Fe(ImP)_2_][PF_6_]) was based on a modified literature synthesis^22,23^. 1,1′-(1,3-phenylene)bis(3-methyl-1-imidazolium) diiodide (1,976 mg; 4 mmol) and tetrakis(dimethylamino)zirconium (1,124 mg; 4.2 mmol) were suspended in dry tetrahydrofuran (20 ml) in a glovebox. The yellow suspension was stirred for 2 h, then iron(ii) bromide (432 mg; 2 mmol) was added. The mixture was stirred for another 16 h. The red/orange mixture was worked up under atmosphere. First, methanol (2 ml) was added. The now blue suspension was stirred under air for 1 h until no further precipitation of a pale solid was observed. The suspension was filtered through a cotton pad and through a porous glass frit. The respective filter cakes were washed with acetonitrile until the filtrate turned colourless. The solvent of the filtrate was evaporated using a rotary evaporator. The blue solid was dissolved in dichloromethane and filtered over a silica column. The column was washed thoroughly with dichloromethane. The blue band was then eluted from the column with acetonitrile. The solvent of the blue fraction was evaporated. The solid was dissolved in methanol (20 ml), and KPF_6_ (2 equiv.; 736 mg; 4 mmol) was added. The desired compound **1** was precipitated by adding water (20 ml) and filtered off. It was redissolved in methanol (50 ml), treated again with KPF_6_ (2 equiv.) and precipitated again with water (50 ml) to ensure a full exchange of the counterion. The suspension was filtered and the blue solid was dried under reduced pressure. It was then dissolved in a minimal amount of dichloromethane and pentane was allowed to diffuse into the solution. Dark blue long needles of **1** were obtained (415 mg; 0.61 mmol; 31%) after crystallization overnight, which were dried under reduced pressure (10^−3^ mbar) for 6 h before elemental analysis and spectroscopic measurements. The results of ^1^H NMR (500 MHz; CD_3_CN) were *δ* = 24.70 (4H), 9.68 (12H), 2.90 (4H), −2.39 (4H) and −35.79 ppm (2H). The electrospray ionization mass spectrometry results for [1-PF_6_]^+^ (C_28_H_26_FeN_8_) were 530.1625 m/z (calculated) and 530.1621 m/z (found). Infrared (attenuated total reflection) analysis of **1** gave measurements of 3,166, 3,141, 2,926, 1,587, 1,470, 1,455, 1,405, 1,344, 1,263, 1,232, 1,075, 874, 824, 769, 714, 682, 555, 394, 354 and 258 cm^−1^. Finally, elemental analysis of **1** (C_28_H_26_F_6_FeN_8_P) gave calculated and found values of 49.80 and 49.76% (C), 3.88 and 4.25% (H) and 16.59 and 16.30% (N), respectively.

The synthesis of [Co(ImP)_2_][PF_6_] (bis(2,6-bis(3-methylimidazol-1-ylidene)phenyl)cobalt(iii) hexafluorophosphate) was analogous to the synthesis of [Fe(ImP)_2_][PF_6_] and was based on the same literature synthesis^22^. 1,1′-(1,3-phenylene)bis(3-methyl-1-imidazolium) diiodide (200 mg; 0.405 mmol) and tetrakis(dimethylamino)zirconium (100 mg; 0.445 mmol) were suspended in dry dichloromethane (10 ml) in a glovebox. The yellow suspension was stirred for 1 h, then cobalt(ii) chloride (26 mg; 0.203 mmol) was added. The mixture was stirred for another 16 h. To quench the reaction, water (1 ml) was added. The suspension was filtered through a cotton pad and a porous glass frit. The respective filter cakes were washed with dichloromethane (10 ml) three times. The red solution was evaporated under reduced pressure. The solid (84 mg; 0.126 mmol) was dissolved in methanol (5 ml), and KPF_6_ (6 equiv.; 140 mg; 0.75 mmol) was added. The compound was precipitated by the addition of water (20 ml) and filtered off. The process was repeated four times to ensure full exchange of the counterion. The suspension was filtered and the light-yellow solid was dried under reduced pressure. It was then dissolved in a minimal amount of acetone and allowed to crystallize under a normal atmosphere. Yellow crystals were obtained (60 mg; 0.0879 mmol; 69% yield). The results of ^1^H NMR (700 MHz; CD_3_CN) were *δ* = 7.72 (d; *J* = 2.0 Hz; 2H), 7.47 – 7.37 (m; 3H), 6.75 (d; *J* = 2.0 Hz; 2H) and 2.52 ppm (s, 6H). The results of ^13^C NMR (706 MHz; CD_3_CN) were *δ* = 207.48, 186.44, 177.78, 149.36, 125.14, 118.31, 116.01, 108.12, 35.86, 30.89 and 1.32 ppm. Finally, elemental analysis of [Co(ImP)_2_][PF_6_] with one molecule of acetone (C_28_H_26_F_6_CoN_8_P × C_3_H_6_O) gave calculated and found values of 50.55 and 50.49% (C), 4.38 and 4.32% (H) and 15.21 and 15.22% (N), respectively.

### X-ray diffraction analysis and crystallographic data deposition

The single-crystal data were recorded using a Bruker SMART CCD area detector diffractometer equipped with a graphite monochromator. The measurements were carried out using Mo *K*α radiation (*λ* = 0.71073 Å) at *T* = 200(2) K, with the temperature variations of the last digit during the measurements in brackets, since at lower temperatures a phase transition occurred, which caused a vaguer diffraction pattern. The structure solution process was carried out following direct methods^46^, and structure refinement was conducted using full-matrix least-squares refinement based on F^2^ (ref. ^46^). All non-H atoms were refined anisotropically. The hydrogen atom positions were derived based on geometry, except the hydrogens of methyl groups. These were located from the Fourier map using HFIX 137 by SHELX^46^. All hydrogen atoms were refined at idealized positions riding on the carbon atoms with isotropic displacement *U*_iso_, which are in case of aromatic carbon atoms 1.2 fold larger than the corresponding displacement parameters of the carbon and in case of hydrogens of a methyl group 1.5 fold larger. The bond lengths of carbon and hydrogen were limited to a range of 0.93 to 0.96 Å. All CH_3_ hydrogen atoms were allowed to rotate but not to tip. One dichloromethane solvent molecule could not be modelled during refinement and was treated using SQUEEZE from the Platon software package^47–49^.

The single crystal of the complex C_28_H_26_N_8_FePF_6_ (molar mass *M*_r_ = 675.40 Da) was obtained in form of a purple block with the size 0.42 × 0.37 × 0.25 mm³. The substance crystallizes in the monoclinic space group *P*2_1_/*c* with a multiplicity *Z* = 4 and the cell parameters *a* = 13.6367(12) Å, *b* = 8.7801(8) Å, *c* = 27.099(3) Å and the angle *β* = 96.285(2)°, resulting in a cell volume *V* = 3225.2(5) Å³, a density *D*_c_ = 1.391 mg/mm^−^^3^and a total number of electrons in the cell F(000) = 1,380; 28,802 reflections of the single crystal were recorded during the measurement to a maximum angle *θ*_max_= 26.462°, whereby 6,622 reflections are independent. This leads to in internal error *R*_int_ = 0.0455 and the refinement converged to a final error R1 = 0.0571 (for all reflections I, which were more intense than two times the standard deviation of the reflexes σ(I)) and to wR2 = 0.1683 (referring to all obtained data). As a result, the maximum residual electron density (with 0.804 eÅ³) is located 0.62 Å from the F-atom F123 and the minimum residual electron density (−0.578 eÅ³) in a distance of 0.61 Å from F123.

Crystallographic data have been deposited in the Cambridge Crystallographic Data Centre (CCDC) database, assigned to the deposition number CCDC 2002774. Copies are available free of charge via www.ccdc.cam.ac.uk.

### Mössbauer spectroscopy and magnetic susceptibility measurements

^57^Fe Mössbauer spectra were recorded with a ^57^Co source in an Rh matrix using an alternating constant acceleration WissEL Mössbauer spectrometer operated in transmission mode and equipped with a Janis closed-cycle helium cryostat. Isomer shifts are given relative to iron metal at ambient temperature. Simulation of the experimental data was performed with the Mfit program using Lorentzian line doublets (E. Bill, Max Planck Institute for Chemical Energy Conversion).

Temperature-dependent magnetic susceptibility measurements were carried out with a Quantum Design MPMS3 SQUID magnetometer in the range from 300 to 2.0 K at a magnetic field of 0.5 T. The powdered sample was contained in a polycarbonate capsule and fixed in a non-magnetic sample holder. Each raw data file for the measured magnetic moment was corrected for the diamagnetic contribution of the sample holder and the capsule. The molar susceptibility data were corrected for the diamagnetic contribution. The simulation of the magnetic data was carried out with the program julX (E. Bill, Max Planck Institute for Chemical Energy Conversion) by applying a fitting procedure to the spin Hamiltonian ${\hat {H}} = g\mu _B\vec B \times \vec S$ for Zeeman splitting of the *S* = ½ spin ground state with Landé factor *g*, the Bohr magneton *μ*_*B*_, the magnetic flux density *B* and the electron spin *S*.

### Cyclic and square wave voltammetry

Dry acetonitrile was obtained by passing HPLC-grade acetonitrile (Fisher) over a column of MP Biomedicals MP Alumina N - Super I, which was activated in an oven at 150 °C for multiple days. The cyclic and square wave voltammetry measurements were performed at room temperature in 0.1 M [(*n*Bu)_4_N][PF_6_] dry acetonitrile with an analyte concentration of 0.001 M under a solvent-saturated argon atmosphere. A three-electrode arrangement with a 1 mm Pt working electrode and a Pt wire counter electrode (both Metrohm) and an Ag/AgCl reference electrode (custom built) was used, with the PGSTAT101 potentiostat from Metrohm. Ferrocene was added after the measurements as an internal standard. All potentials were referenced against the FcH^0/+^ couple. The voltammograms were analysed using the NOVA software (version 2.1.3). The diagnostic criteria for reversibility were based on those proposed by Nicholson^50,51^ and the Randles–Sevcik^52,53^ equation.

### Absorption spectroscopy (steady state)

Acetonitrile of spectroscopic grade (SPECTRONORM VWR Acetonitrile) was used as the solvent for steady-state absorption spectroscopy.

Steady-state absorption spectra were recorded using solutions with concentrations of ~10^−5^–10^−4^ M in quartz cuvettes (path length = 10 mm) using a Cary 50 or PerkinElmer Lamda 45 spectrometer.

### Theoretical calculations

Quantum chemical calculations were performed at D_2d_ symmetry with DFT and linear response TDDFT using the optimally tuned long-range separation functional LC-BLYP together with a combined basis set: def2TZVP (Fe) and 6-311 G(d,p) (all other atoms). Tuning of the functional was done using the so-called delta self-consistent field method^54–56^. The details can be found in the work of Bokarev et al.^28^. The following parameters were obtained for the present complex: *α* = 0 (that is, the percentage of the exact exchange in the short range) and 0.15 Bohr^-1^ (long-range separation parameter). Solvent effects (acetonitrile) were taken into account within the polarized continuum model approach^57^. Calculations were done with the G16 (ref. ^58^) and Q-Chem 5.3 (ref. ^59^) packages. Excited-state analysis was performed using the TheoDORE package^60^. Analysis of Huang–Rhys factors, tuning of the functional and generation of geometries along normal modes were done with in-house codes. Further information on the calculations can be found in the Supplementary Information.

### Fragment-based excited-state analysis

To consider the localization of excitations involving ligand orbitals in a more precise way, excited-state analysis with the TheoDORE package was performed for the molecule split into five moieties: a central iron atom, two phenyl unities and two imidazole unities. All possible types of excitation were considered (see Supplementary Fig. 11). The resulting analysis is shown in Supplementary Fig. 12. As one can see, the excitations within the LMCT band are spread over the entire ligand, with a slightly dominant imidazole unity. In turn, the MLCT band also consists of the excitations to the entire ligand.

### Spectroelectrochemistry

Ultraviolet/visible/near-infrared spectroelectrochemical experiments were performed using a BioLogic SP-50 voltammetric analyser and a Specac Omni-Cell liquid transmission cell with CaF_2_ windows equipped with a Pt gauze working electrode, a Pt gauze counter electrode and an Ag wire as the pseudo reference electrode, melt-sealed in a polyethylene spacer (approximate path length = 1 mm) in CH_3_CN containing 0.1 M [*n*Bu_4_N][PF_6_]^61^.

### Room temperature emission spectroscopy

For steady-state emission spectroscopy, acetonitrile of spectroscopic grade was used as the solvent.

Steady-state emission spectra were recorded in 10 mm quartz cuvettes on an Edinburgh Instruments FLS1000 spectrometer with single monochromators and a red-extended PMT-980 detector or a HORIBA Scientific FluoroMax-4 spectrofluorometer. The solutions for the measurements under argon were degassed using the freeze–pump–thaw technique.

For determination of the Stokes shift, the highest-intensity features of the respective absorption and emission bands were used. Nonetheless, excitation at lower energies, such as 410 and 630 nm, also led to the observation of the respective MLCT and LMCT emission bands.

### Variable-temperature emission spectroscopy

Variable-temperature emission spectra were recorded on a Varian Cary Eclipse spectrometer. For low-temperature photoluminescence measurements, a solution of the complex in butyronitrile (refluxed over Na_2_CO_3_ and KMnO_4_, distilled and stored over aluminium oxide) was filled into a quartz cuvette in an argon-filled glovebox and the cuvette was sealed and transferred to an Oxford cryostat (Oxford instruments OptistatDN). Measurements were conducted between 297 and 87 K.

### Femtosecond transient absorption spectroscopy

Femtosecond transient absorption spectra were recorded using excitation wavelengths in three different optical regions and thereby somewhat different pump–probe setups. In all cases, they were based on regenerative Ti:sapphire laser systems operating at a frequency of 1 kHz and at a centre wavelength of either 775 nm (CPA 2001; Clark MXR) or 800 nm (Spitfire Pro; Spectra-Physics). For probing, a white light continuum generated by focusing a small fraction of the Ti:sapphire output into a CaF_2_ crystal was used. Pump and probe beams were focused onto the sample to overlapping spots with diameters in the range of 200–400 µm for the pump and of 100 µm for the probe. The polarizations of the pump and probe pulses were set to the magic angle with respect to each other. After the sample, the probe was dispersed by a prism and transient absorption changes were spectrally resolved and recorded by an array detector.

For pumping the sample with an excitation wavelength of 400 nm, the output of the Ti:sapphire system (Spitfire Pro) was frequency doubled using a beta barium borate (BBO) crystal. The resulting time resolution was ~150 fs.

To obtain ultrashort excitation pulses in the visible range with a centre wavelength of 600 nm, a non-collinear optical parametric amplifier (NOPA) pumped by the Ti:sapphire system (CPA 2001) was applied. The dispersion of the NOPA pulses was minimized by a compressor based on fused silica prisms, resulting in an overall time resolution of better than 100 fs.

For excitation in the ultraviolet range (that is, at a centre wavelength of 330 nm), the NOPA was tuned to 660 nm and its output was frequency doubled by a 100-µm-thick BBO crystal cut for type I phase matching.

For all measurements, the iron complex was dissolved in acetonitrile under argon and the sample solution was filled into a fused silica cuvette with a thickness of 1 mm.

The obtained data were fitted using a global fit. In the global fit, the multi-exponential model function $F\left( {\lambda ,t} \right)\!=\!\mathop {\sum }\limits_i^N {\rm{DAAS}}_i\left( \lambda \right)\!\times\!{{{\mathrm{exp}}}}( - {{{t}}}/\tau _i)$, convoluted with the temporal response of the pump–probe setup, was fitted to the complete set of time-dependent transient absorption spectra, yielding the decay associated amplitude spectra DAAS_i_ and the associated time constants $\tau _i$. In the present case, three exponential decay components were necessary to reproduce the data with satisfying accuracy (that is, *N* = 3).

### SCMs

To investigate the time-resolved luminescence, a streak camera (Streakscope C10627; Hamamatsu Photonics) was applied. The samples were prepared and measured under argon in 1 cm cuvettes. For excitation at 388 nm, ultrashort laser pulses generated by frequency doubling the output of a Ti:sapphire laser system (CPA 2001; Clark MXR) were used. For excitation at 330 nm, a NOPA was set to a centre wavelength of 660 nm and its output pulses were frequency doubled using a BBO crystal. To ensure that only radiation at 330 nm reached the sample, a fused silica prism was applied to separate the ultraviolet pulses from the fundamental.

The luminescence lifetimes were determined by fitting a monoexponential decay to the data in the spectral region 640–840 nm and a double exponential decay to the data of the region 390–600 nm.

Averaged lifetimes *τ*_av_ were obtained using:

$$\tau _{{\rm{av}}} = \frac{{A_1 \times \tau _1 + A_2 \times \tau _2}}{{A_1 + A_2}}$$

With *A*_1_ and *A*_2_ being the integrals and *τ*_1_ and *τ*_2_ being the lifetimes of the respective amplitude spectra.

### TCSPC and time-resolved emission spectroscopy

Time-resolved emission spectroscopy (TRES) was employed through TCSPC using a HORIBA Ultima 01-DD (HORIBA Jobin Yvon). The degassed sample solution was excited at 374 nm using a HORIBA DD375L laser diode with a maximum repetition rate of 100 MH. The emission was recorded in 5-nm steps between 440 and 520 nm in reverse mode with a slit of 6 nm of the emission monochromator. Repetitive start–stop signals were recorded using a multi-channel analyser over the course of 1 h per wavelength. A histogram of photons was recorded as a function of 16,383 channels on a time range of 100 ns (0.012 ns per channel). To evaluate the TRES data, a global fitting procedure was applied to the data, similar to the analysis of the transient absorption data.

### Quenching experiments

The experimental setup for the quenching experiments was the same as in the SCMs. The quencher was one-sixth of the solution, resulting in a concentration of 1.0 M for triethanolamine, 1.6 M for benzonitrile and 2.3 M for triethylamine.

### Singlet oxygen sensitization

**DPBF** was used to indirectly detect the generation of ^1^O_2_^62,63^. The reaction was carried out in air-saturated MeOH solutions at room temperature. For the reactions, 2.4 ml of a **DPBF** solution (20 µM) was mixed with 0.5 ml solutions of either **1** (10^−4^ M), [Ru(bpy)_3_]^2+^ (10^−4^ M) or pure MeOH in a Hellma 1-cm-path fluorescence cuvette. The reaction mixtures were illuminated in an Edinburgh Instruments FLS1000 fluorescence spectrophotometer at 350 nm with a slit of 2.5 nm. The decomposition of DPBF was detected by monitoring the luminescence intensity at 450 nm after excitation at 350 nm. The emission spectra were recorded at 5-min intervals with a dwell time of 0.2 s in the range of 420–600 nm.

## Online content

Any methods, additional references, Nature Portfolio reporting summaries, source data, extended data, supplementary information, acknowledgements, peer review information; details of author contributions and competing interests; and statements of data and code availability are available at 10.1038/s41557-023-01137-w.

### Supplementary information

Supplementary Information Supplementary Figs. 1–21 and Tables 1 and 2.

Supplementary Data 1 DFT-calculated geometries as .xyz files and in a PDF.

Supplementary Data 2 Crystallographic data for compound **1** (CCDC reference 2002774).

### Source data

Source Data Fig. 1 Mössbauer data.

Source Data Fig. 2 Electrochemical and absorption data.

Source Data Fig. 3 Emission and excitation spectra and transient absorption and streak camera data.

## Data Availability

Crystallographic data have been deposited in the CCDC database, assigned to the deposition number CCDC 2002774. Copies are available free of charge via www.ccdc.cam.ac.uk. Source data are provided with this paper.

## Code availability

The codes used for the analysis of Huang–Rhys factors, functional tuning and the generation of geometries along normal modes is available from O.K. (oliver.kuehn@uni-rostock.de) upon reasonable request.
