# Supplementary material for: Janus-type emission from a cyclometalated iron(iii) complex
Source: Nat Chem. 2023 Feb 27;15(4):468–74. doi: 10.1038/s41557-023-01137-w (PMC10070185; doi:10.1038/s41557-023-01137-w)
Supplement: Supplementary file 1 — Supplementary Figs. 1–21 and Tables 1 and 2. [file 41557_2023_1137_MOESM1_ESM.pdf]

nature chemistry

Article

https://doi.org/10.1038/s41557-023-01137-w

Janus-type emission from a cyclometalated iron(III) complex

In the format provided by the authors and unedited

Supplementary Information for

Janus-type Emission from a Cyclometalated Iron(III) Complex

Jakob Steube[1], Ayla Kruse[2], Olga S. Bokareva[3,4], Thomas Reuter[5], Serhiy Demeshko[6], Roland Schoch[1], Miguel A. Argüello Cordero[2], Athul Krishna[1], Stephan Hohloch[7], Franc Meyer[6], Katja Heinze[5], Oliver Kühn[2], Stefan Lochbrunner[2], Matthias Bauer[1*]

1 Institute of Inorganic Chemistry and Center for Sustainable Systems Design (CSSD), Paderborn University, Warburger Straße 100, 33098 Paderborn, Germany

2 Institute of Physics and Department for Life, Light and Matter, University of Rostock, 18051 Rostock, Germany

3 Institute of Physics, University of Rostock, 18051 Rostock, Germany

4 Institute of Physics, University of Kassel, Heinrich-Plett-Str. 40, 34132, Kassel, Germany

5 Department of Chemistry, Johannes Gutenberg University, Duesbergweg 10–14, 55128 Mainz, Germany

6 Institute of Inorganic Chemistry, University of Göttingen, Tammannstrasse 4, 37077 Göttingen, Germany

7 Institute of General, Inorganic and Theoretical Chemistry, University of Innsbruck, Innrain 80-82, 6020 Innsbruck, Austria

Content

Synthesis, NMR and mass spectrometry 1
X-ray diffraction analysis and crystallographic data deposition 4
Mößbauer spectroscopy and magnetic susceptibility measurements 6
Cyclic and square wave voltammetry 7
Absorption spectroscopy (steady-state) 8
Theoretical Calculations 9
Spectroelectrochemistry 18
Room temperature emission spectroscopy 20
Variable temperature emission spectroscopy 22
Femtosecond transient absorption spectroscopy 23
Time correlated single photon counting and time resolved emission spectroscopy .. 25
Quenching experiments 26
Singlet oxygen sensitization 27

Synthesis, NMR and mass spectrometry

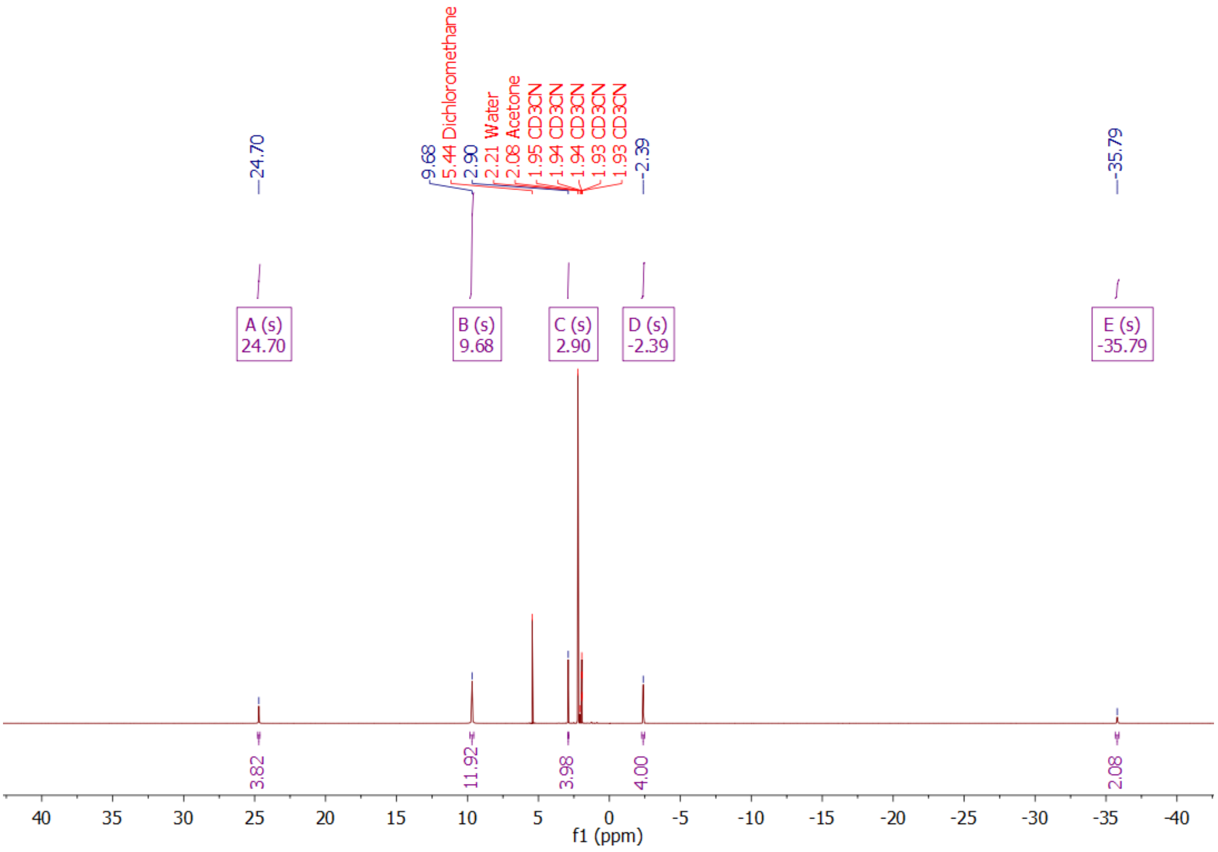

Supplementary Figure 1| ^{1}H NMR characterization of [Fe(ImP)$_2$][PF$_6$]. The spectrum was recorded in deuterated acetonitrile on a scan range from -50 – 50 ppm. The spectrum shows the expected number of resonances and integrals of **1** (26 H nuclei in total) and resonances of the residual solvents.

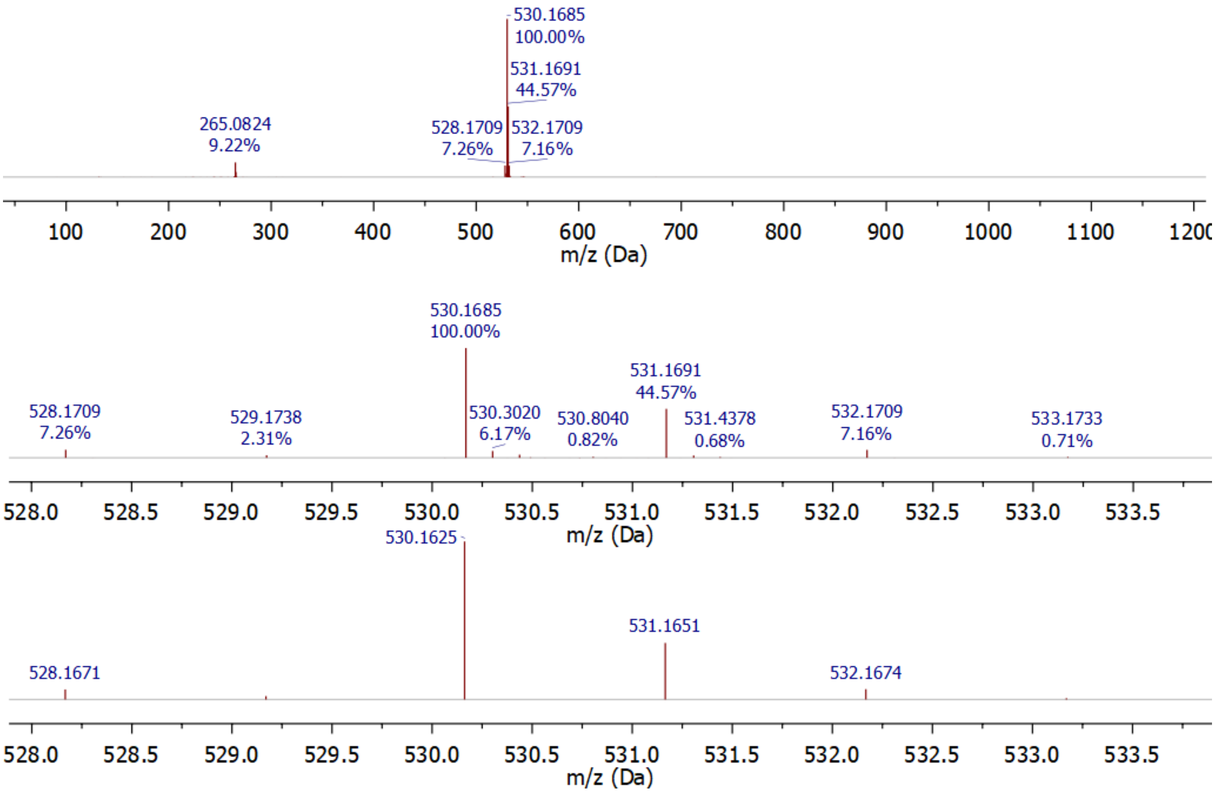

Supplementary Figure 2| ESI mass spectrometry measurement of [Fe(ImP)$_2$][PF$_6$]. Top: Full spectrum over the whole measurement range, showing [M-PF$_6$]$^+$ at 530.1685 m/z and the ESI-oxidized [M-PF$_6$]$^{2+}$ species at 265.0824 m/z. Middle: Enlargement on the [M-PF$_6$]$^+$ range. Bottom: Simulation of [M-PF$_6$]$^+$ shows good agreement to the experimental isotopic pattern.

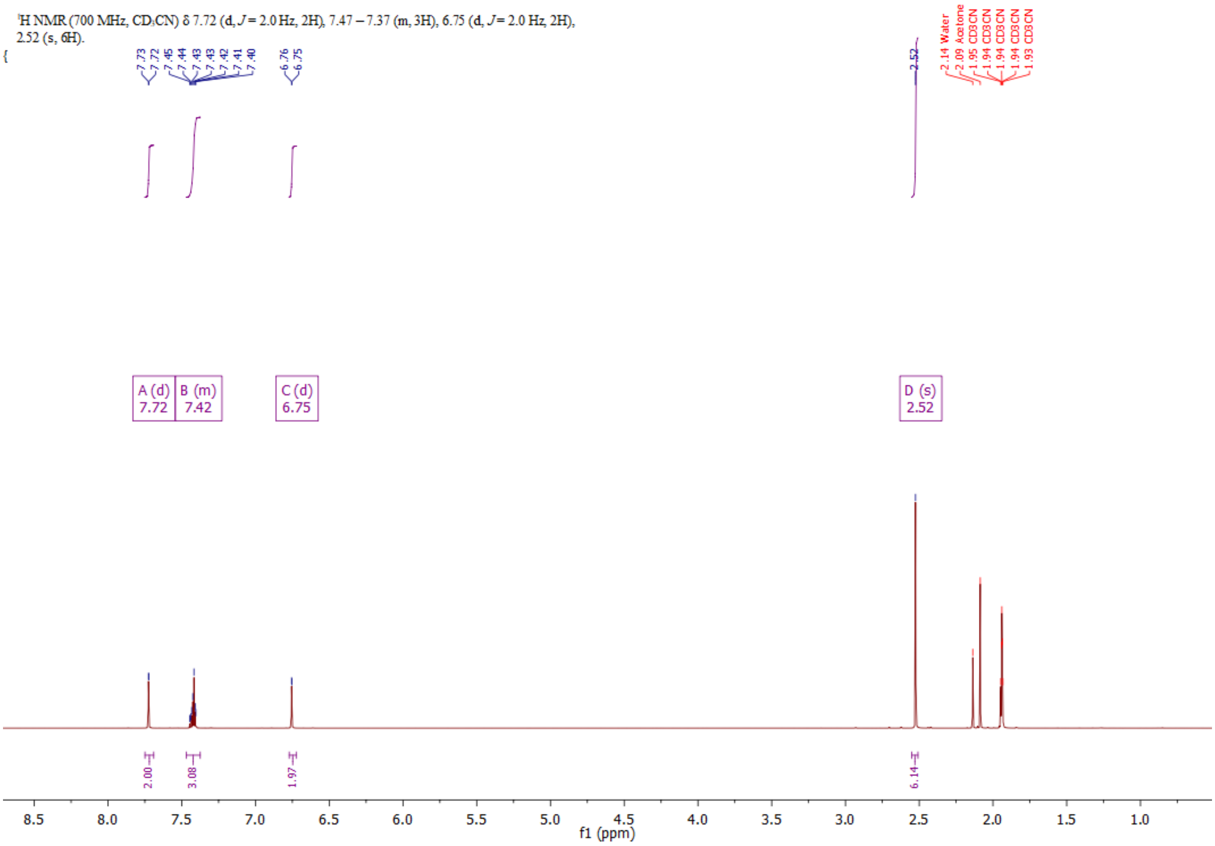

Supplementary Figure 3| ^{1}H NMR characterization of [Co(ImP)$_2$][PF$_6$]. The spectrum was recorded in deuterated acetonitrile. The spectrum shows the expected number of resonances and integrals (13 H nuclei in total for the half complex of [Co(ImP)$_2$][PF$_6$]).

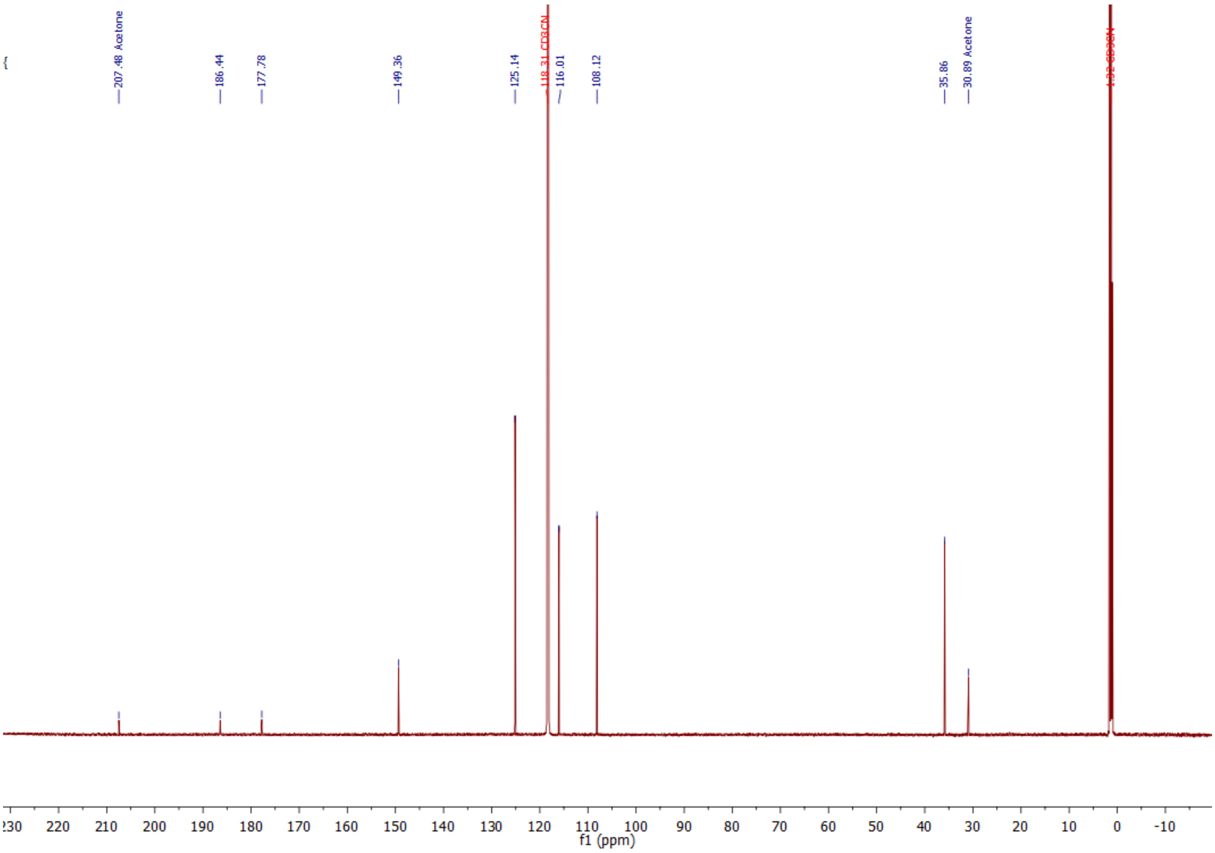

Supplementary Figure 4| ^{13}C NMR characterization of [Co(ImP)$_2$][PF$_6$]. The spectrum was recorded in deuterated acetonitrile. The spectrum shows the expected number of resonances and resonances of the residual solvent.

X-ray diffraction analysis and crystallographic data deposition

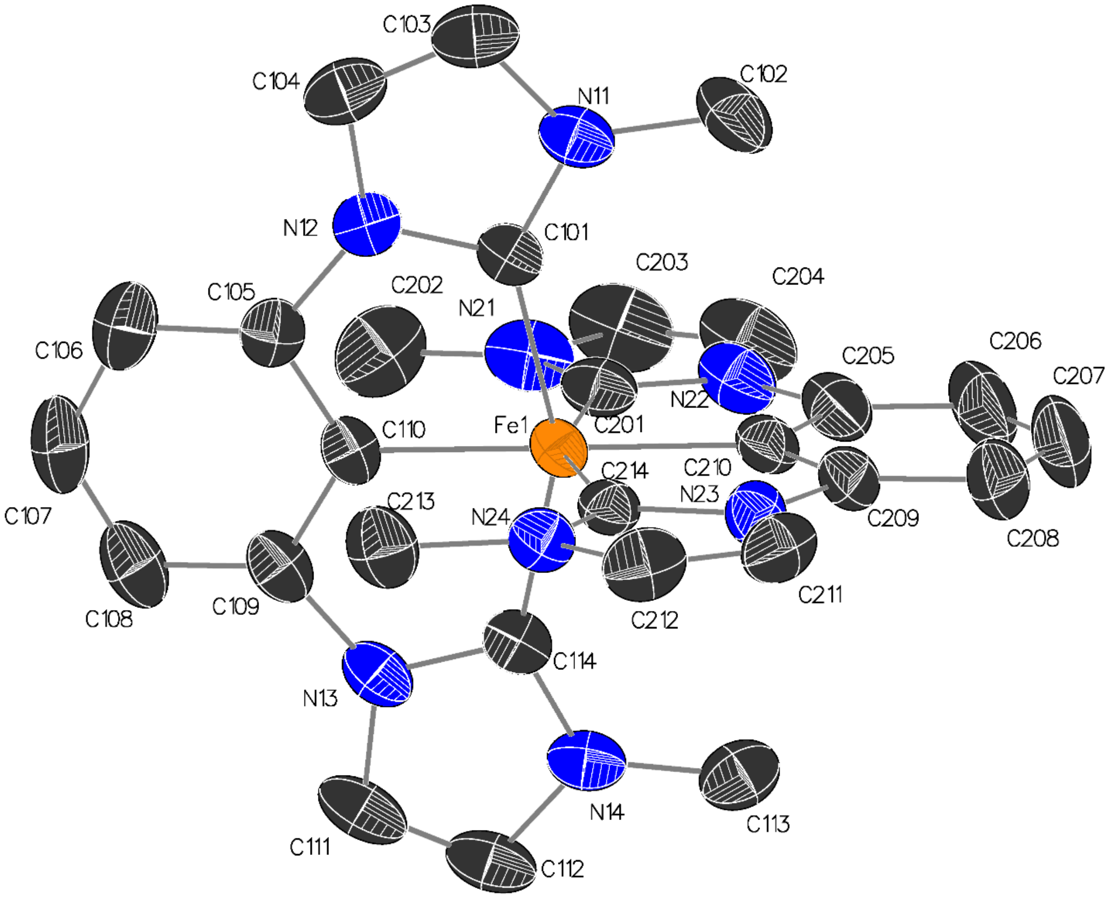

Supplementary Figure 5| Ortep plot of [Fe(ImP)$_2$][PF$_6$]. Structure of the cation of **1** as determined by X-ray diffraction, hydrogen atoms and counter ion are omitted for clarity. Crystallographic data have been deposited at the Cambridge Crystallographic Data Centre, assigned to the deposition number CCDC 2002774. Copies are available free of charge via www.ccdc.cam.ac.uk.

Mößbauer spectroscopy and magnetic susceptibility measurements

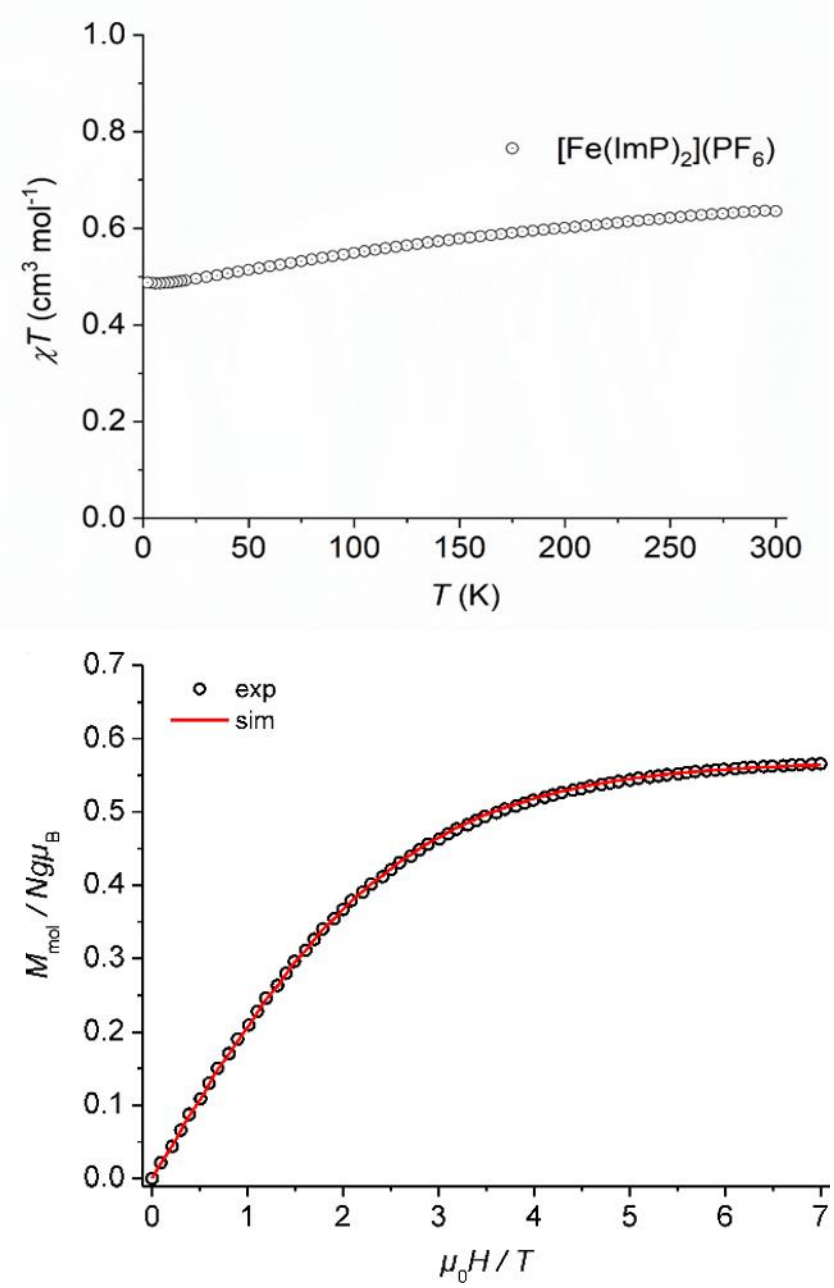

Supplementary Figure 6| Magnetic characterization of [Fe(ImP)$_2$][PF$_6$]. Top: Magnetic susceptibility data as the temperature dependence of $\chi_M T$ in the range of 2 – 300 K at 0.5 T, showing the typical behavior of metal complexes with $(t_{2g})^5$ electron configuration ($^2T_2$) with significantly higher $\chi_M T$ values than expected for a conventional S = ½ spin system (spin-only) due to spin-orbit coupling. Bottom: Plot of the magnetization vs. field at 2 K. The simulation was performed for S = ½ with g = 2.28 (fixed).

Cyclic and square wave voltammetry

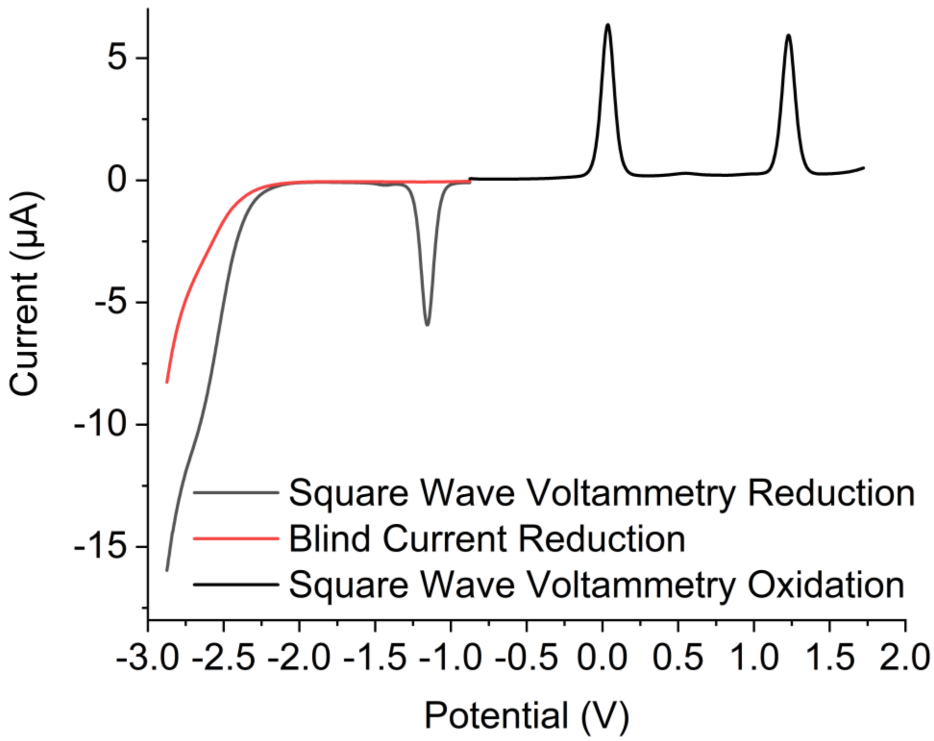

Supplementary Figure 7| Square wave voltammetry of [Fe(ImP)$_2$][PF$_6$]. Square wave voltammogram of **1** in CH_3CN/[*n*Bu$_4$N][PF$_6$] of the reduction up to -2.85 V vs FcH$^{0/+}$ compared with the blind current during that process at a scan rate of 100 mV s^{-1}. This indicates that the ligand reduction is observed at around -2.7 V.

Absorption spectroscopy (steady-state)

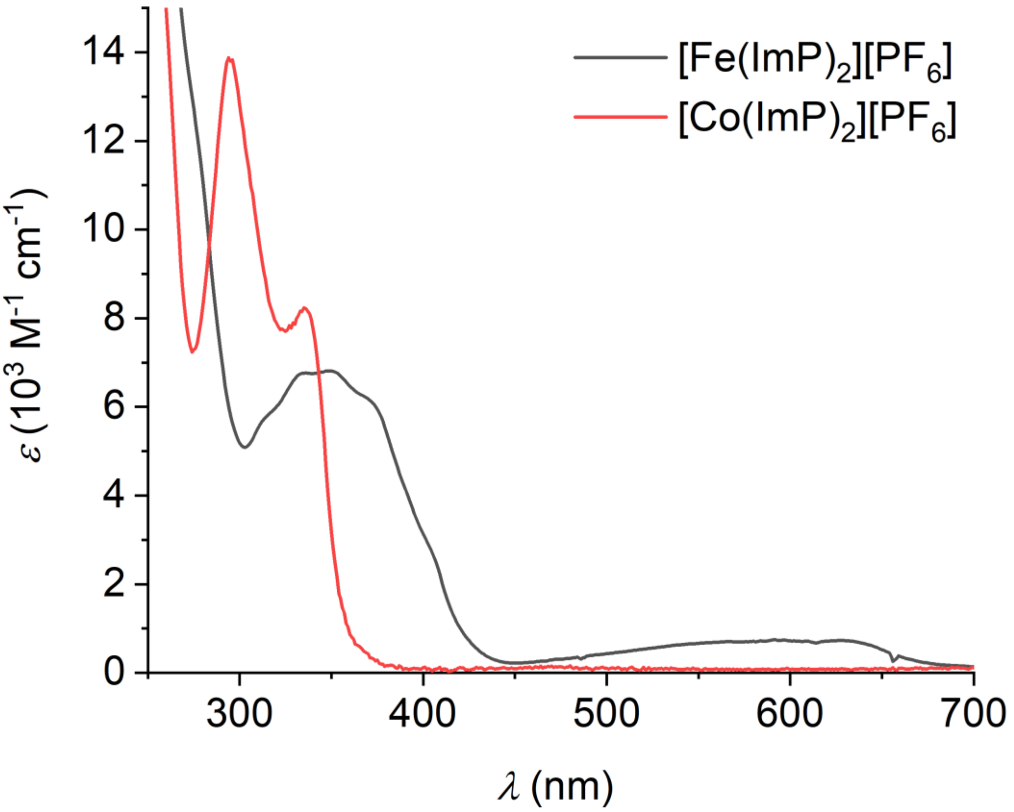

Supplementary Figure 8| Absorption spectra of $[Fe(ImP)_2][PF_6]$ and $[Fe(ImP)_2][PF_6]$. Absorption spectra in the region between 250 nm and 700 nm.

Theoretical Calculations

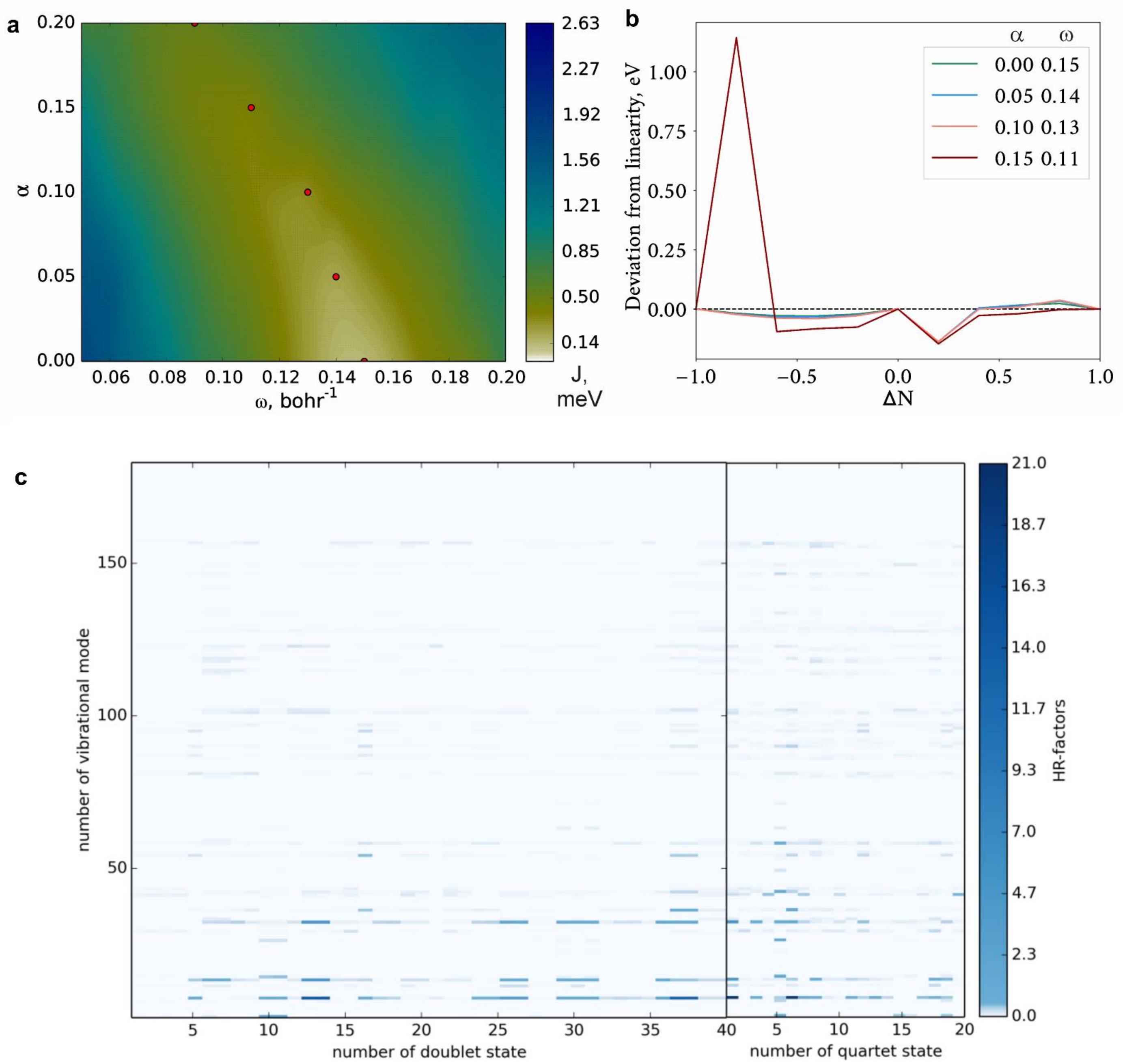

Supplementary Figure 9| Theoretical investigations of [Fe(ImP)$_2$][PF$_6$]. a,b, Tuning of the LC-BLYP functional for DFT calculations. **a,** functional $J^*(\alpha, \omega) = \sqrt{J_0^2 + J_1^2}$, $J_0 = |\varepsilon_{HOMO}(N) + IP(N)|$ and $J_1 = |\varepsilon_{HOMO}(N+1) + EA(N)|$ showing the deviation from Koopman's theorem for ionization potential (IP) and electron affinity (EA) for the system with N electrons. The red points denote the minima of the J^*-functional at constant α values. **b,** the deviation from linearity of energy dependence between integer number of electrons. As a result of tuning, the parameters α=0.0 and ω=0.15 Bohr^{-1} were found. **c,** Huang-Rhys factors for the lowest 40 doublet and 20

quartet states as calculated using TDDFT. The mode shown in Fig. 4 of the main text corresponds to the mode with index 7 (ω/(2πc)=126 cm^{-1}). It has the largest Huang-Rhys factor among the lowest 15 doublet states.

Supplementary Table 1: Main orbital densities in **1**$^{-}$, **1,** and **1**$^{+}$.

Orb. Nr.	**1**$^{-}$ Fe^{II} (singlet)		**1** Fe^{III} (doublet)				**1**$^{+}$ Fe^{IV} (triplet)			
	Orbital (↑↓)		Orbital (α, ↑)		Orbital (β, ↓)		Orbital (α, ↑)		Orbital (β, ↓)	
136	HOMO-2	Fe (t_{2g})	SOMO-2	Fe (t_{2g})	SOMO-1	Fe (t_{2g})	SOMO-2	Ligand	SOMO	Fe (t_{2g})
137	HOMO-1	Fe (t_{2g})	SOMO-1	Fe (t_{2g})	SOMO	Fe (t_{2g})	SOMO-1	Ligand	SUMO	Fe (t_{2g})
138	HOMO	Fe (t_{2g})	SOMO	Fe (t_{2g})	SUMO	Fe (t_{2g})	SOMO	Fe (t_{2g})*	SUMO+1	Fe (t_{2g})
139	LUMO	Ligand	SUMO	Ligand	SUMO+1	Ligand	SUMO	Ligand	SUMO+2	Ligand
140	LUMO+1	Ligand	SUMO+2	Ligand	SUMO+2	Ligand	SUMO+2	Ligand	SUMO+3	Ligand

Orbital densities were calculated on the respective optimized structure (**1**$^{-}$, **1,** and **1**$^{+}$ as singlet, doublet, and triplet, respectively) to show the metal character of the reduction and oxidation. In the three doubly occupied HOMOs, the density is mainly located on the iron. In the oxidized species (**1**$^{+}$), the density of the α-orbitals is mainly delocalized on the ligands with partial Fe-fraction. The β-orbitals show more of a "t_{2g}-behavior", with the SOMO and the SUMO/SUMO+1 (singly occupied/unoccupied molecular orbital) orbital representing the t_{2g}-orbitals, indicating metal-based oxidation.

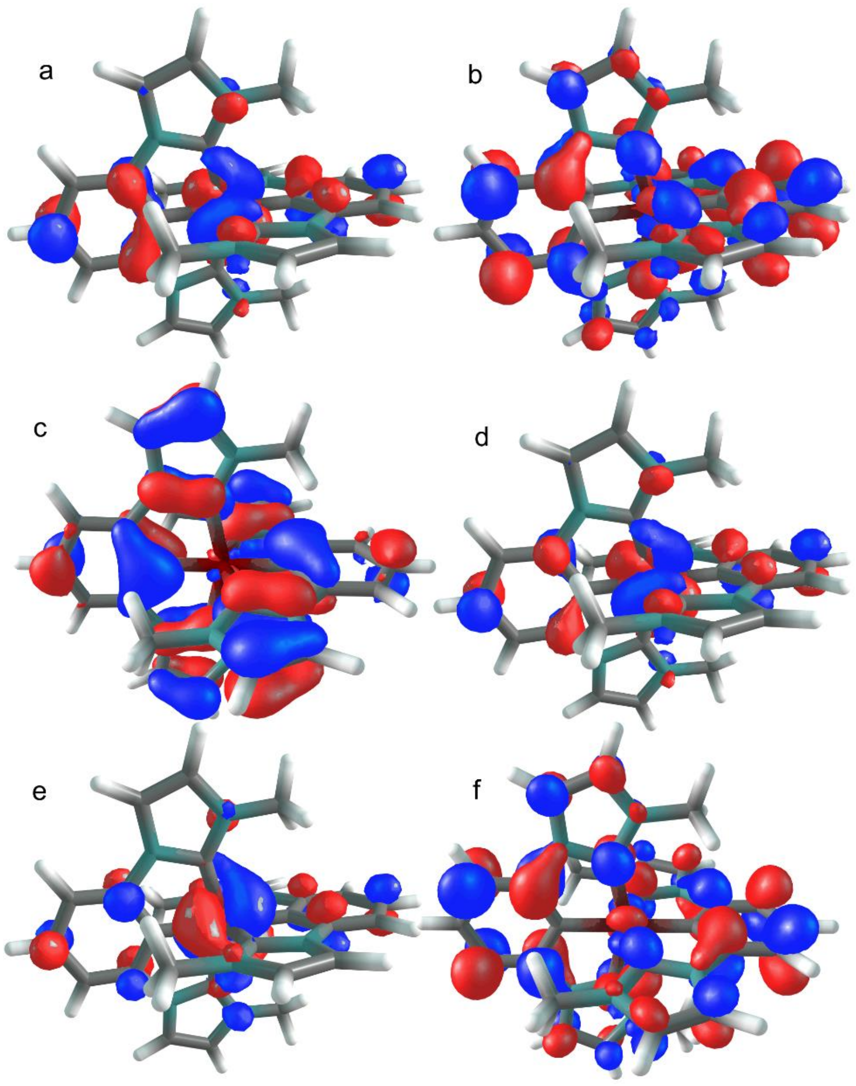

Supplementary Figure 10| Orbitals involved in the electronic transitions. a, Orbital a138. **b,** Orbital a139. **c,** Orbital b135. **d,** Orbital b137. **e,** Orbital b138. **f,** Orbital b139.

Supplementary Table 2: Vertical excitation energies predicted with tuned LC-BLYP. Note that only the most important contributions are presented, and therefore the sum of weights for a given state may be less than 1. The letters S and V describe the singly (S) and vacant (V) orbitals.

No.	E, eV	Osc.str.	Weight	Involved orbitals				
1	0.18	0.0023	1.03	137	→	S	1	β
2	0.26	0.0009	1.01	136	→	S	1	β
3	1.88	0.0009	0.85	131	→	S	1	β
			0.13	134	→	S	1	β
4	1.97	0.0115	0.96	135	→	S	1	β
17	3.43	0.0012	0.38	127	→	S	1	β
			0.15	136	→	V	4	β
			0.15	137	→	V	2	β
			0.11	137	→	V	7	β
18	3.45	0.0018	0.63	137	→	V	4	β

5	2.08	0.0000	0.97	133	→	S	1	β
6	2.24	0.0000	0.86	134	→	S	1	β
			0.13	131	→	S	1	β
7	2.46	0.0056	0.97	132	→	S	1	β
8	2.68	0.0164	0.89	129	→	S	1	β
9	2.85	0.0000	0.88	128	→	S	1	β
10	2.89	0.0110	0.97	130	→	S	1	β
11	3.09	0.0000	0.63	136	→	V	1	β
			0.20	137	→	V	1	α
12	3.10	0.0040	0.80	137	→	V	1	β
			0.09	S1	→	V	1	α
13	3.24	0.0043	0.62	137	→	V	2	β
			0.15	137	→	V	7	β
			0.08	S1	→	V	5	α
			0.06	S1	→	V	2	α
14	3.31	0.0112	0.60	136	→	V	2	β
			0.14	136	→	V	7	β
			0.08	137	→	V	2	α
			0.07	137	→	V	5	α
15	3.39	0.0000	0.68	137	→	V	3	α
			0.16	S1	→	V	4	α
16	3.40	0.0025	0.85	124	→	S	1	β
			0.11	S1	→	V	3	α
			0.06	136	→	V	7	β
19	3.48	0.0096	0.27	S1	→	V	5	α
			0.23	127	→	S	1	β
			0.18	137	→	V	7	β
			0.12	137	→	V	2	β
20	3.52	0.0117	0.52	S1	→	V	1	α
			0.28	136	→	V	3	β
			0.10	137	→	V	1	β
21	3.53	0.0187	0.29	137	→	V	5	α
			0.24	136	→	V	7	β
			0.21	136	→	V	2	β
			0.10	137	→	V	4	β
22	3.62	0.0280	0.50	136	→	V	3	β
			0.26	S1	→	V	1	α
23	3.64	0.0040	0.60	136	→	V	4	β
			0.29	127	→	S	1	β
24	3.66	0.0018	0.74	126	→	S	1	β
			0.17	123	→	S	1	β
25	3.67	0.0000	0.46	137	→	V	1	α
			0.20	136	→	V	1	β
			0.09	S1	→	V	4	α
26	3.70	0.0129	0.46	137	→	V	1	α
			0.20	136	→	V	1	β
			0.09	S1	→	V	4	α
			0.07	134	→	V	1	α
			0.06	136	→	V	7	β
27	3.71	0.0384	0.65	S1	→	V	2	α
			0.15	136	→	V	1	α
			0.06	137	→	V	2	β
28	3.74	0.0007	0.33	137	→	V	9	β
			0.17	S1	→	V	6	α
			0.15	137	→	V	6	β
			0.08	S1	→	V	8	α
			0.07	137	→	V	13	β
29	3.86	0.0861	0.53	S1	→	V	3	α
			0.11	137	→	V	2	α
			0.11	137	→	V	4	β
30	3.86	0.0008	0.80	122	→	S	1	β
			0.10	125	→	S	1	β
31	3.88	0.0338	0.70	137	→	V	3	α
			0.07	136	→	V	4	β
32	3.92	0.0000	0.21	137	→	V	6	α
			0.19	136	→	V	6	β
			0.15	136	→	V	9	β
			0.05	136	→	V	5	β
34	3.97	0.0000	0.18	136	→	V	9	β
			0.11	132	→	V	1	α
			0.10	137	→	V	8	α
			0.09	134	→	V	2	β
			0.08	134	→	V	2	α
			0.08	137	→	V	1	α
			0.08	132	→	V	1	β
35	4.00	0.0232	0.36	137	→	V	5	α
			0.28	136	→	V	7	β
			0.10	S1	→	V	3	α
36	4.01	0.0003	0.68	120	→	S	1	β
			0.08	136	→	V	5	α
			0.06	136	→	V	2	α
			0.05	137	→	V	4	α
37	4.06	0.0071	0.34	137	→	V	4	α
			0.09	135	→	V	1	α
			0.08	120	→	S	1	β
			0.08	136	→	V	2	α
			0.08	135	→	V	1	β
38	4.06	0.0040	0.25	136	→	V	1	α
			0.15	S1	→	V	2	α
			0.12	135	→	V	2	α
			0.09	135	→	V	2	β
			0.08	133	→	V	1	β

33	3.93	0.0097	0.38	137	→	V	4	α
			0.12	135	→	V	1	α
			0.11	136	→	V	3	β
			0.09	136	→	V	2	α
			0.06	135	→	V	1	β
			0.05	132	→	V	2	α
			0.05	135	→	V	3	α
40	4.13	0.0000	0.53	S1	→	V	4	α
			0.11	137	→	V	3	β
			0.05	136	→	V	1	β
41	4.18	0.0000	0.20	125	→	S	1	β
			0.15	136	→	V	3	α
			0.08	135	→	V	4	α
			0.06	135	→	V	3	β
42	4.20	0.0008	0.43	S1	→	V	5	α
			0.31	137	→	V	7	β
43	4.21	0.0209	0.20	123	→	S	1	β
			0.10	132	→	V	2	α
			0.08	132	→	V	2	β
			0.07	136	→	V	4	α
			0.07	134	→	V	1	α
			0.06	134	→	V	1	β
			0.05	137	→	V	2	α
			0.05	137	→	V	5	β
			0.05	126	→	S	1	β
			0.05	S1	→	V	7	α
44	4.23	0.0001	0.32	S1	→	V	6	α
			0.13	137	→	V	9	β
			0.10	136	→	V	2	α
			0.08	S1	→	V	8	α
			0.06	136	→	V	5	α
			0.05	135	→	V	1	β
45	4.27	0.0198	0.34	136	→	V	1	α
			0.11	133	→	V	1	α
			0.05	135	→	V	3	α
53	4.52	0.0098	0.27	137	→	V	5	β
			0.13	136	→	V	12	β
			0.07	137	→	V	6	β
			0.06	137	→	V	12	α
			0.05	135	→	V	3	α
54	4.54	0.0008	0.04	130	→	V	2	α
			0.11	137	→	V	6	α
			0.11	136	→	V	3	α
			0.11	133	→	V	3	α
			0.05	136	→	V	6	β
			0.05	133	→	V	4	β

			0.07	137	→	V	3	α
39	4.07	0.0020	0.21	137	→	V	2	α
			0.10	137	→	V	5	α
			0.10	S1	→	V	3	α
			0.07	136	→	V	4	α
			0.10	133	→	V	1	β
			0.09	135	→	V	2	β
46	4.29	0.0144	0.22	137	→	V	6	β
			0.16	S1	→	V	8	α
			0.12	136	→	V	2	α
			0.06	137	→	V	5	β
			0.05	133	→	V	2	α
47	4.33	0.0000	0.17	136	→	V	6	β
			0.12	137	→	V	8	α
			0.09	125	→	S	1	β
			0.05	132	→	V	1	α
48	4.37	0.0096	0.24	137	→	V	9	β
			0.14	137	→	V	6	β
			0.14	136	→	V	2	α
			0.07	135	→	V	1	β
49	4.37	0.0227	0.49	123	→	S	1	β
			0.13	126	→	S	1	β
			0.09	137	→	V	5	β
			0.06	136	→	V	4	α
50	4.39	0.0000	0.53	125	→	S	1	β
			0.12	136	→	V	3	α
51	4.48	0.0073	0.43	136	→	V	5	β
			0.12	136	→	V	6	β
			0.07	136	→	V	11	β
52	4.49	0.0058	0.15	137	→	V	5	β
			0.08	136	→	V	12	β
			0.07	134	→	V	1	α
			0.05	130	→	V	1	β
			0.05	130	→	V	1	α
			0.06	132	→	V	4	α
58	4.60	0.0221	0.30	131	→	V	1	α
			0.14	136	→	V	4	α
			0.13	131	→	V	1	β
			0.07	136	→	V	12	β
59	4.61	0.0048	0.51	131	→	V	1	α
			0.11	136	→	V	4	α
60	4.62	0.0007	0.27	136	→	V	10	β
			0.07	137	→	V	10	α
			0.07	135	→	V	1	α
61	4.63	0.0000	0.31	137	→	V	8	β
			0.24	137	→	V	10	β

55	4.54	0.0103	0.13	133	→	V	1	β
			0.12	135	→	V	2	β
			0.09	134	→	V	4	α
			0.09	137	→	V	7	α
			0.08	132	→	V	3	α
			0.06	135	→	V	2	α
			0.05	133	→	V	1	α
56	4.56	0.0000	0.45	121	→	S	1	β
			0.08	134	→	V	3	α
			0.07	132	→	V	4	α
			0.05	132	→	V	3	β
			0.05	136	→	V	10	β
57	4.57	0.0129	0.19	135	→	V	1	β
			0.18	135	→	V	1	α
			0.07	133	→	V	2	β
			0.07	133	→	V	2	α
			0.07	137	→	V	6	β
66	4.72	0.0001	0.08	134	→	V	3	α
			0.50	136	→	V	5	α
			0.08	120	→	S	1	β
			0.05	136	→	V	2	α
67	4.74	0.0000	0.45	131	→	V	2	α
			0.28	131	→	V	2	β
68	4.76	0.0442	0.15	134	→	V	4	α
			0.14	136	→	V	5	β
			0.10	137	→	V	12	β
			0.09	134	→	V	3	β
			0.08	132	→	V	3	α
			0.06	132	→	V	4	β
69	4.79	0.0000	0.46	131	→	V	2	β
			0.28	131	→	V	2	α
			0.05	134	→	V	2	β
70	4.82	0.0303	0.24	131	→	V	3	α
			0.11	S1	→	V	8	α
			0.10	136	→	V	8	β
			0.08	131	→	V	4	β
			0.08	S1	→	V	6	α
			0.06	136	→	V	2	α
			0.05	134	→	V	3	α
71	4.82	0.0005	0.21	136	→	V	4	α
			0.10	S1	→	V	7	α
			0.06	133	→	V	4	α
			0.05	128	→	V	4	α
			0.05	136	→	V	9	α
72	4.83	0.0001	0.11	137	→	V	8	β
			0.09	136	→	V	3	α

			0.05	136	→	V	3	α
62	4.64	0.0008	0.19	133	→	V	1	α
			0.11	136	→	V	11	β
			0.10	135	→	V	2	α
			0.05	137	→	V	11	α
63	4.64	0.0001	0.41	121	→	S	1	β
			0.18	136	→	V	8	β
			0.08	135	→	V	1	α
			0.07	136	→	V	5	α
			0.05	136	→	V	10	β
64	4.66	0.0395	0.70	131	→	V	1	β
			0.06	131	→	V	1	α
			0.05	136	→	V	4	α
65	4.67	0.0001	0.32	137	→	V	6	α
			0.19	136	→	V	9	β
			0.11	136	→	V	3	α
			0.06	137	→	V	8	α
			0.08	136	→	V	7	α
			0.06	131	→	V	2	β
73	4.84	0.0095	0.34	131	→	V	3	α
			0.21	S1	→	V	8	α
			0.12	S1	→	V	6	α
			0.10	131	→	V	4	β
74	4.84	0.0097	0.51	136	→	V	6	α
			0.14	136	→	V	8	α
75	4.88	0.0029	0.36	137	→	V	12	β
			0.22	131	→	V	4	α
			0.19	131	→	V	3	β
76	4.88	0.0589	0.28	S1	→	V	7	α
			0.22	134	→	V	1	α
			0.09	135	→	V	4	β
			0.06	134	→	V	1	β
			0.06	133	→	V	3	β
77	4.89	0.0507	0.18	131	→	V	4	α
			0.14	119	→	S	1	β
			0.10	137	→	V	12	β
			0.09	131	→	V	3	β
			0.07	135	→	V	2	α
			0.07	137	→	V	7	α
			0.07	136	→	V	5	β
78	4.91	0.0187	0.20	136	→	V	8	β
			0.13	S1	→	V	8	α
			0.09	S1	→	V	6	α
			0.09	131	→	V	3	α
			0.06	134	→	V	3	α
			0.06	135	→	V	1	β

79	4.92	0.0000	0.24	137	→	V	10	β
			0.09	131	→	V	2	α
			0.06	136	→	V	6	β
			0.05	136	→	V	9	β
			0.05	131	→	V	5	α
80	4.93	0.0015	0.16	137	→	V	12	β
			0.15	135	→	V	2	α
			0.10	131	→	V	4	α
			0.06	136	→	V	8	α
			0.06	135	→	V	5	α
			0.05	131	→	V	3	β
81	4.96	0.0123	0.13	135	→	V	1	α
			0.10	134	→	V	4	β
			0.09	129	→	V	1	β
			0.06	136	→	V	10	β
			0.05	135	→	V	1	β
			0.05	136	→	V	8	β
			0.05	129	→	V	1	α
82	4.97	0.0000	0.22	137	→	V	8	β
			0.21	137	→	V	10	β
			0.16	131	→	V	5	α
			0.09	137	→	V	8	α
83	4.98	0.2584	0.31	S1	→	V	7	α
			0.19	135	→	V	3	α
			0.12	134	→	V	1	β
			0.06	136	→	V	12	β
84	4.99	0.0321	0.73	119	→	S	1	β
			0.05	137	→	V	7	α
85	5.02	0.0000	0.13	S1	→	V	9	α
			0.07	137	→	V	6	α
			0.06	134	→	V	2	α
			0.06	131	→	V	5	α
			0.06	134	→	V	2	β
			0.05	132	→	V	1	β
			0.05	137	→	V	8	β
93	5.12	0.0109	0.22	137	→	V	7	α
			0.10	132	→	V	3	α
			0.07	129	→	V	2	β
			0.05	135	→	V	2	α
			0.05	130	→	V	4	α
			0.05	131	→	V	3	β
			0.05	128	→	V	1	β
94	5.13	0.0007	0.22	131	→	V	5	α
			0.12	137	→	V	8	α
			0.08	S1	→	V	9	α
			0.08	135	→	V	4	α

			0.13	134	→	V	2	α
			0.11	137	→	V	10	β
			0.10	S1	→	V	10	α
			0.09	137	→	V	8	β
			0.09	131	→	V	5	α
			0.06	132	→	V	1	β
			0.06	135	→	V	3	β
86	5.04	0.0049	0.85	137	→	V	11	β
87	5.05	0.0021	0.27	133	→	V	2	α
			0.17	133	→	V	2	β
			0.14	135	→	V	1	β
			0.10	135	→	V	1	α
			0.06	133	→	V	5	α
88	5.06	0.0185	0.32	133	→	V	1	α
			0.23	137	→	V	7	α
			0.12	135	→	V	2	α
89	5.06	0.0010	0.32	131	→	V	4	β
			0.14	134	→	V	4	β
			0.12	134	→	V	3	α
			0.09	131	→	V	3	α
			0.06	132	→	V	3	β
90	5.08	0.0021	0.43	133	→	V	1	β
			0.40	135	→	V	2	β
91	5.10	0.0030	0.32	131	→	V	3	β
			0.23	131	→	V	4	α
			0.13	134	→	V	3	β
			0.09	134	→	V	4	α
92	5.11	0.0000	0.10	137	→	V	8	α
			0.08	134	→	V	5	α
			0.06	S1	→	V	7	α
97	5.15	0.0001	0.76	137	→	V	13	β
			0.12	137	→	V	9	β
98	5.15	0.0002	0.58	135	→	V	5	α
			0.06	134	→	V	3	β
			0.05	136	→	V	8	α
99	5.17	0.0624	0.05	131	→	V	6	α
			0.23	135	→	V	3	α
			0.16	136	→	V	12	β
			0.12	135	→	V	4	β
			0.07	134	→	V	1	α
			0.05	137	→	V	12	α
100	5.18	0.0001	0.14	136	→	V	13	β
			0.14	135	→	V	4	α
			0.12	132	→	V	1	α
			0.09	S1	→	V	9	α
			0.07	131	→	V	7	β

			0.07	S1	→	V	10	α
			0.06	131	→	V	7	β
95	5.13	0.0199	0.22	131	→	V	4	β
			0.15	133	→	V	2	β
			0.12	133	→	V	2	α
			0.09	131	→	V	3	α
			0.07	135	→	V	1	β
			0.07	132	→	V	3	β
96	5.14	0.1789	0.13	134	→	V	1	α
			0.22	136	→	V	12	β
			0.10	134	→	V	1	β
			0.09	137	→	V	12	α
			0.06	131	→	V	5	α
			0.05	132	→	V	1	β
			0.05	136	→	V	9	β

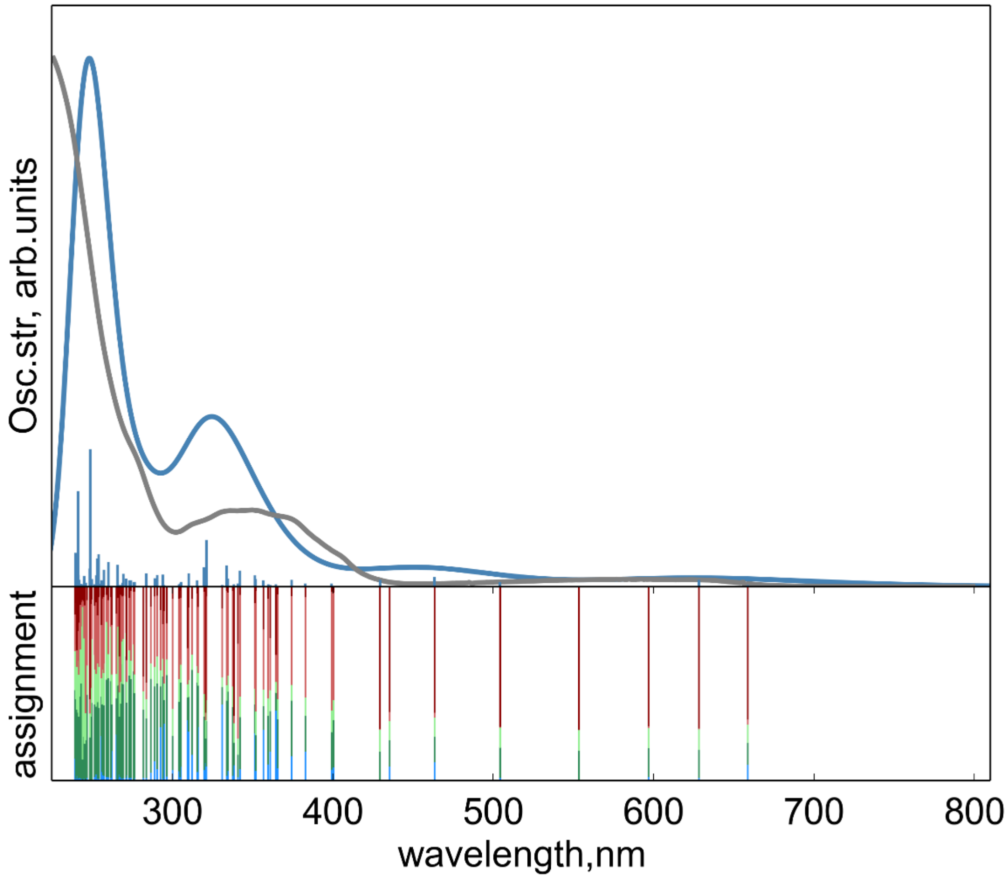

Supplementary Figure 11| Overlay of the simulated optical absorption spectrum (blue line, method: tuned LC-BLYP) and the experimental spectrum (grey line). Broadening: Gaussian line shape, σ=0.2 eV.

Fragment-based excited-states analysis

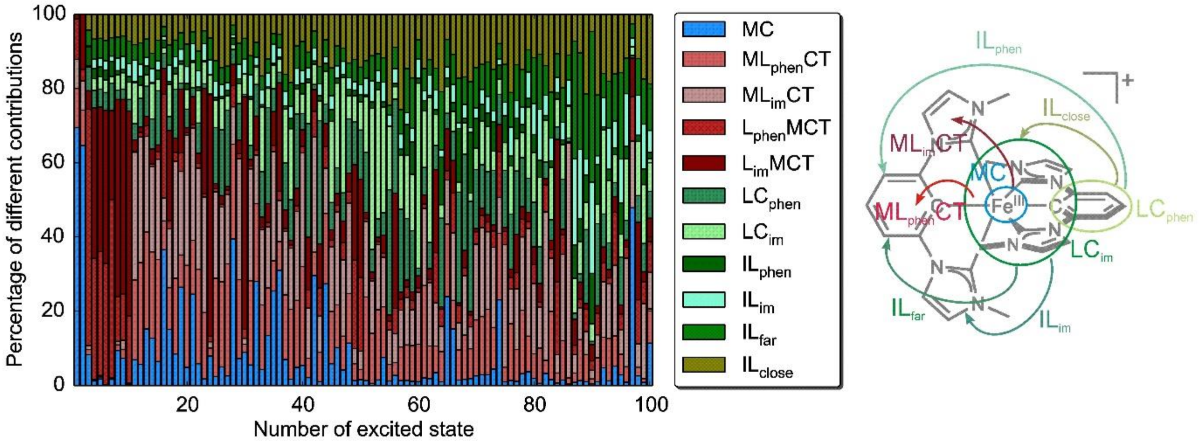

Supplementary Figure 12| Density-matrix analysis of the lowest TDDFT transitions based on the 5 fragments presented in the right part of the figure.

Spectroelectrochemistry

a

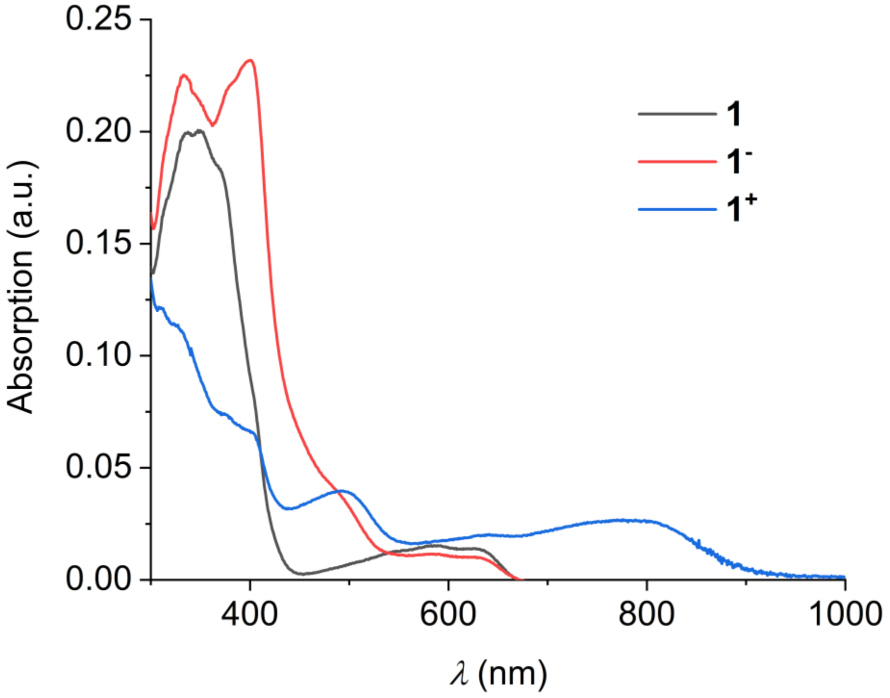

b

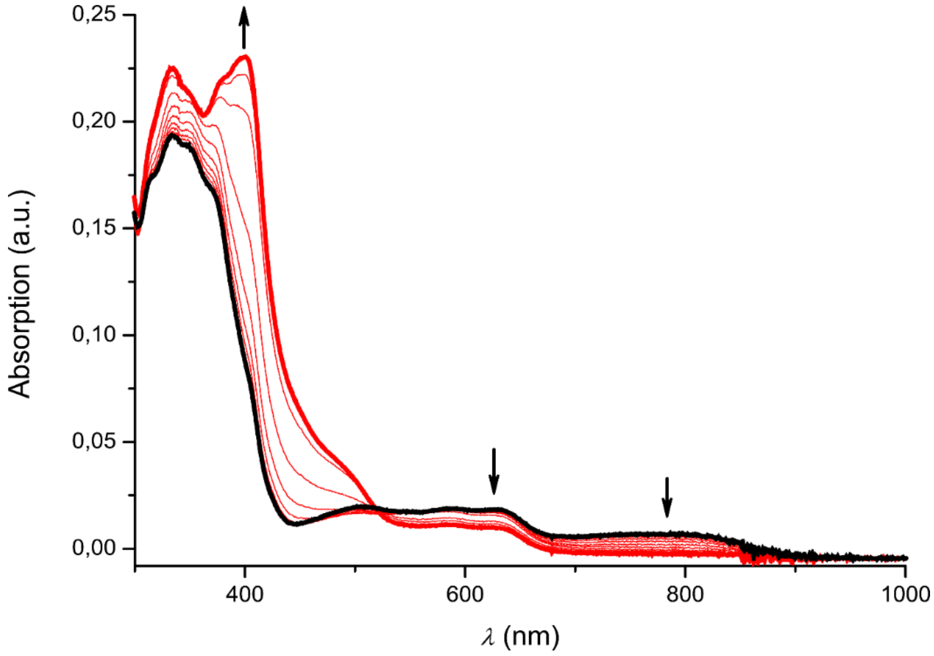

c

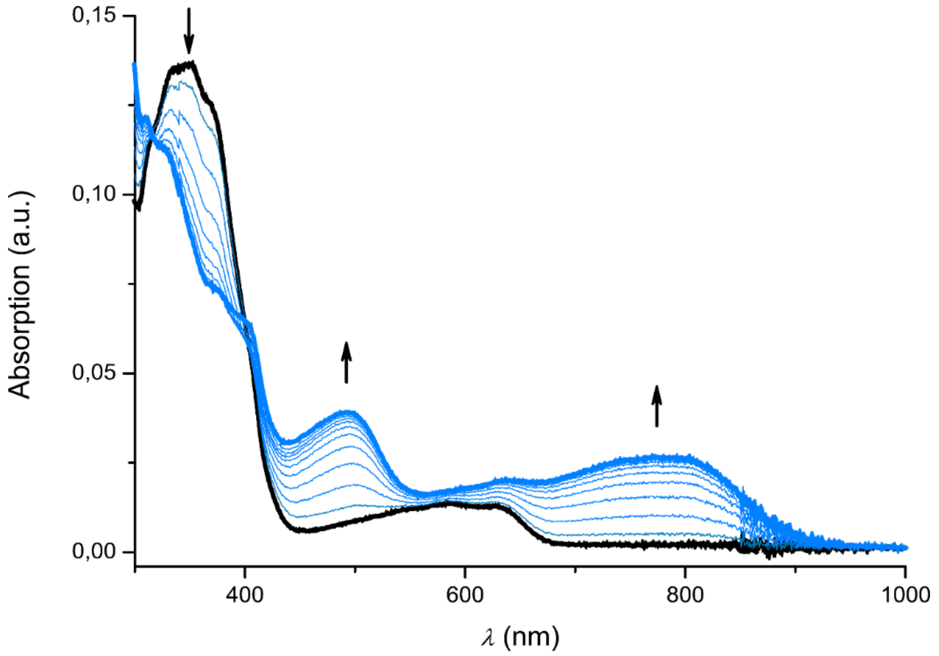

Supplementary Figure 13| Spectroelectrochemical investigations. a) UV-Vis/NIR absorption spectrum of **1** (grey) in comparison with that of $\mathbf{1}^{-}$ (red) and $\mathbf{1}^{+}$ (blue) obtained by spectro-electrochemical measurements in CH_3CN/[*n*Bu_4N][PF_6]. b) Spectra acquired during oxidative SEC. c) Spectra acquired during reductive SEC.

Room temperature emission spectroscopy

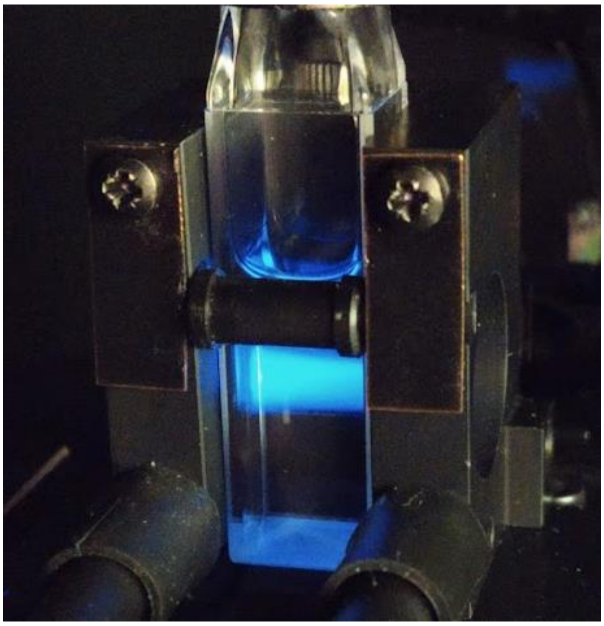

Supplementary Figure 14| Room temperature emission. Observed emission after excitation at 350 nm in degassed acetonitrile.

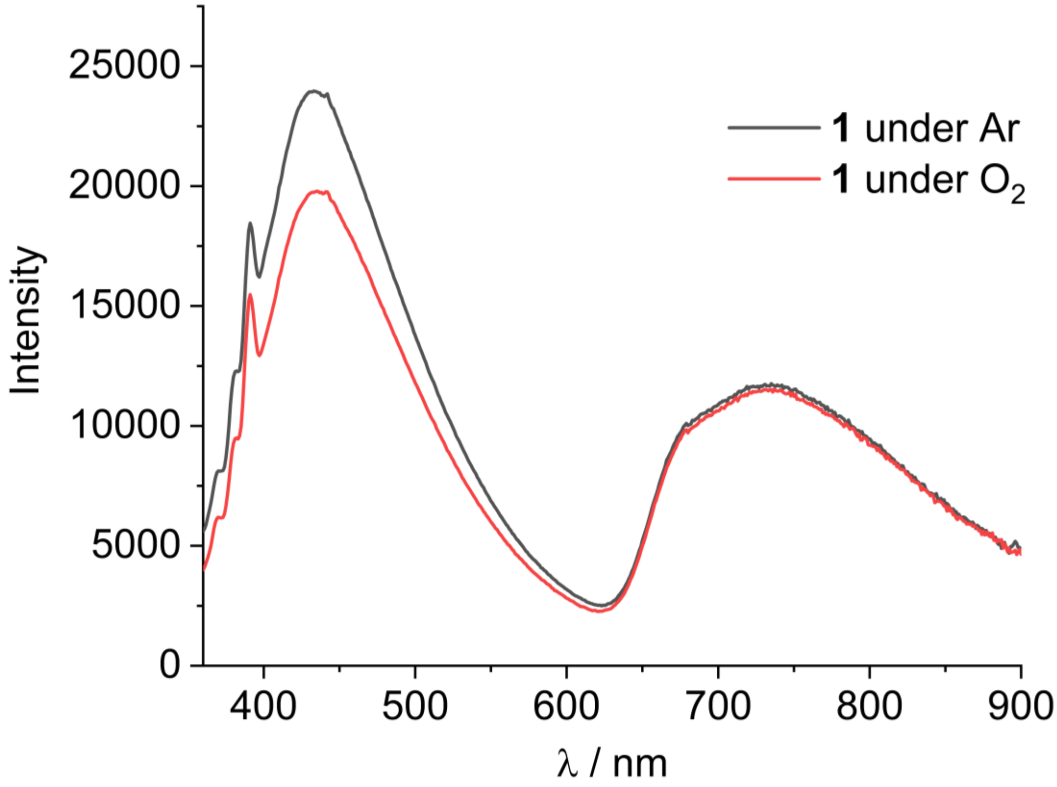

Supplementary Figure 15| Room temperature emission. Emission after excitation at 350 nm in degassed acetonitrile and after being re-aerated under atmosphere.

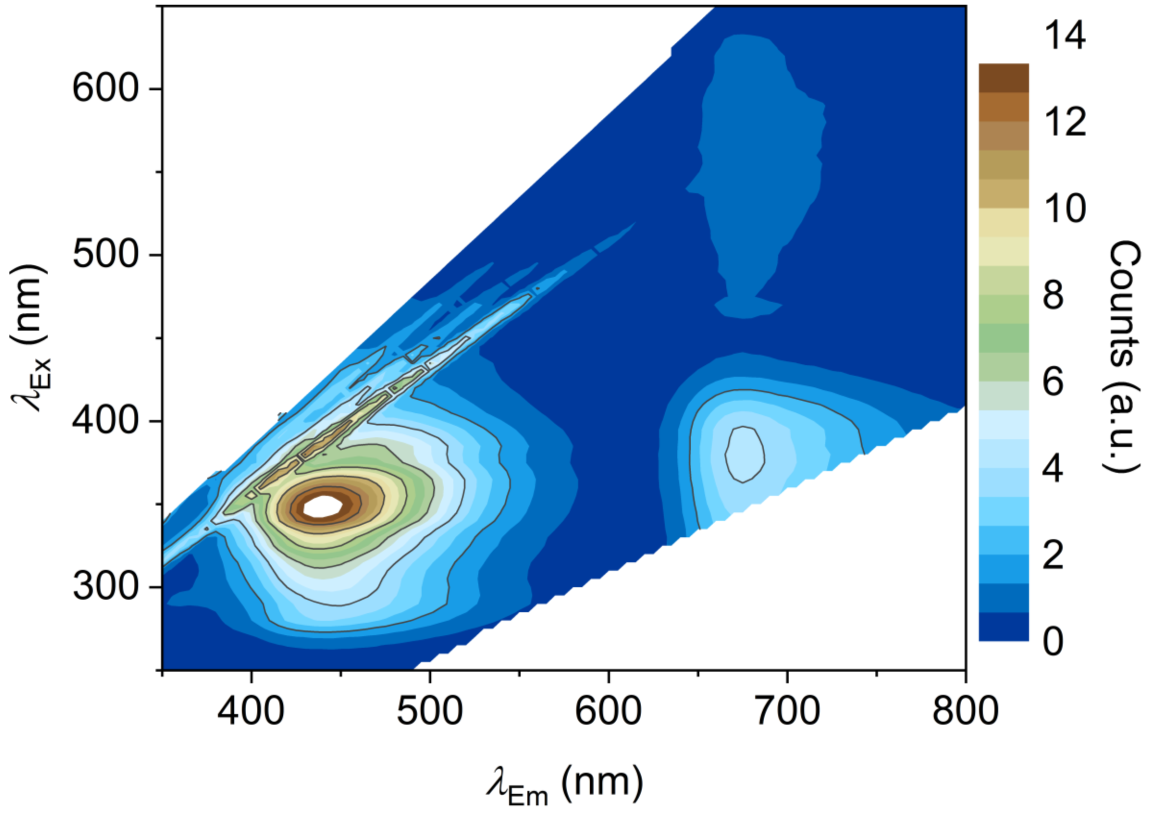

Supplementary Figure 16| Excitation-emission Spectra. Excitation-emission plot for **1**. The plot shows the separation between the MLCT and the LMCT fluorescence. Excitation in the CT-absorption band (310 – 410 nm) shows both MLCT and LMCT emissions. Above this wavelength, the LMCT emission is visible. The narrow diagonal features below 600 nm are caused by Raman signals from the solvent. In this measurement, no correction for the detector sensitivity in the red was employed. Hence the maximum of the LMCT emission is at 675 nm instead of 735 nm.

For the determination of the Stokes shift, the highest intensity features of the respective absorption and emission bands were used. Nonetheless, excitation at lower energies, such as 410 and 630 nm, also led to the observation of the respective MLCT and LMCT emission bands.

Variable temperature emission spectroscopy

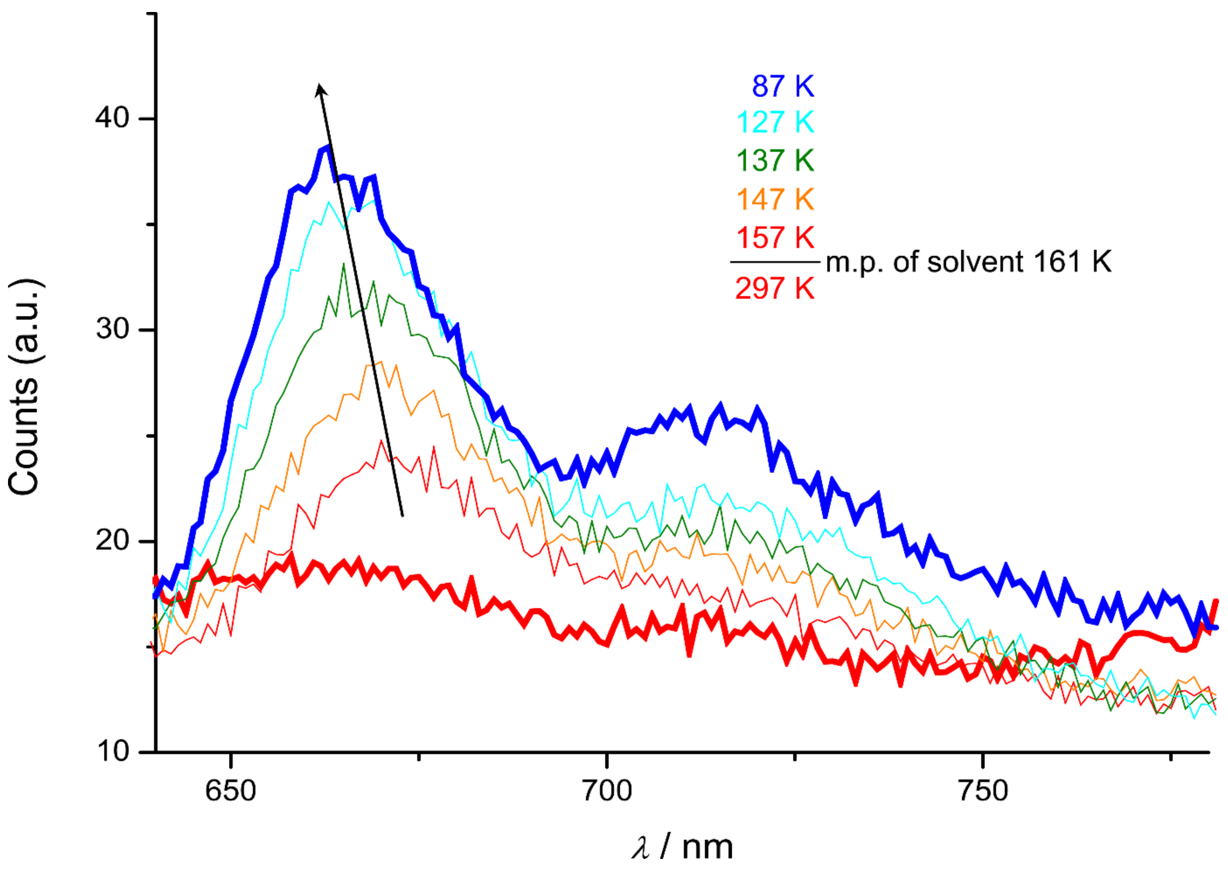

Supplementary Figure 17| Variable Temperature Emission. The spectra of **1** were collected in butyronitrile with an excitation wavelength of 425 nm, showing the intensity increase upon glass formation and further cooling and the blue shift of the LMCT band upon cooling. With this excitation wavelength and with the employed detector, the luminescence in fluid solution is barely visible.

Femtosecond transient absorption spectroscopy

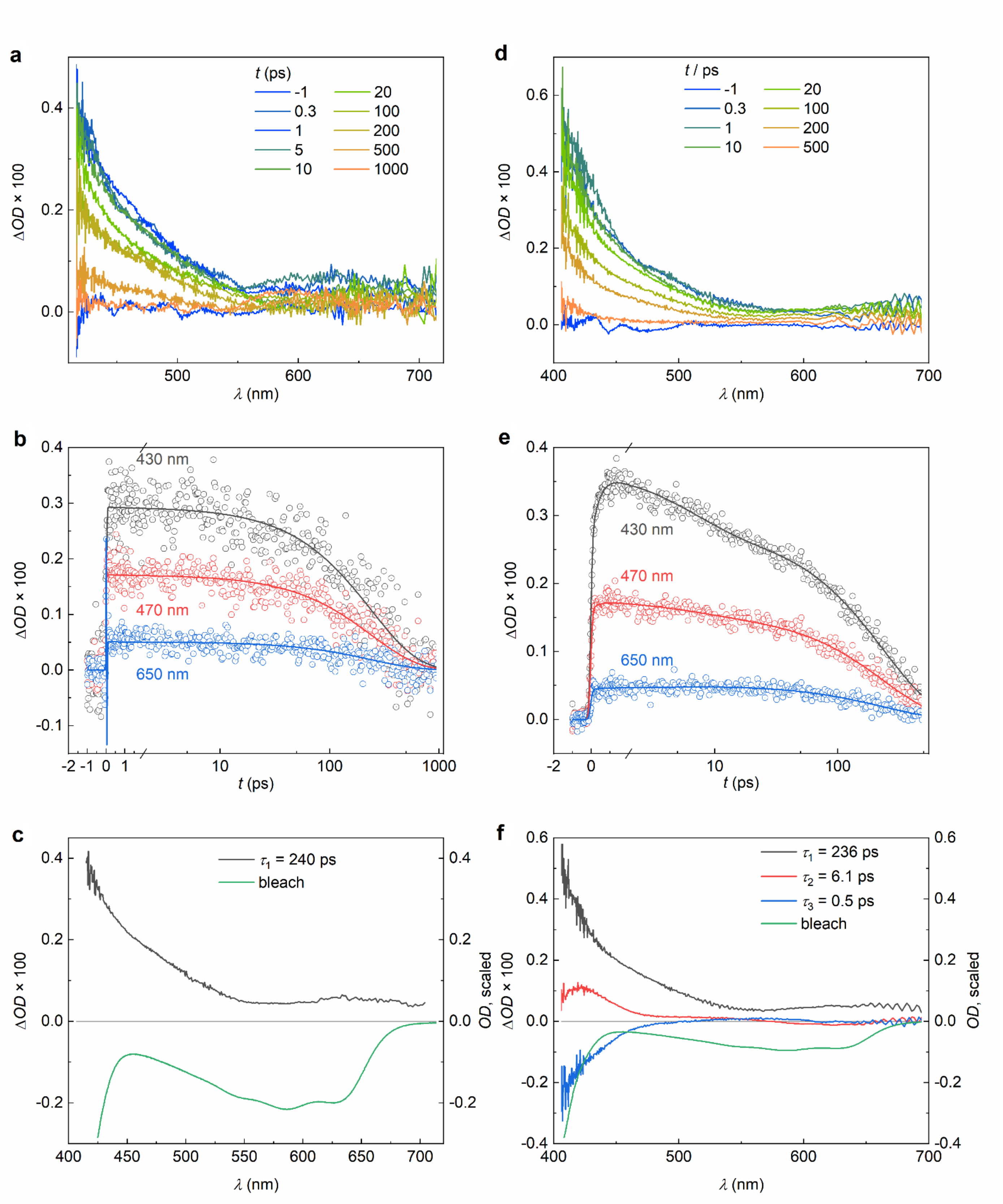

Supplementary Figure 18| Transient absorption (TA) spectra with pump pulses centered at 600 nm and 330 nm, respectively. a, b, c TA data for excitation of complex **1** at 600 nm and corresponding fits. A degassed acetonitrile solution of **1** was used as sample. A global fit, as described in the manuscript, was applied and a pure monoexponential decay with a time constant of τ_1 = 240 ps was found. **a,** TA spectra at the given times after excitation at 600 nm. **b,** Time traces of the transient absorption of **1** at three selected detection wavelengths together with the time curves resulting from the global fit. **c,** Decay associated amplitude spectrum of the single exponential decay in comparison with the ground state bleach, i. e., the scaled negative absorption spectrum. **d, e, f** TA data for excitation of complex **1** at 330 nm and corresponding fits. **d,** TA spectra obtained at the given times after excitation of a degassed solution of **1** in acetonitrile at 330 nm. **e,** Time traces of the transient absorption of **1** at three selected detection wavelengths together with the time curves resulting from the global fit. **f,** Decay associated amplitude spectra for the three components with τ_1 = 236 ps, τ_2 = 6.1 ps, and τ_3 = 0.5 ps in comparison with the ground state bleach, i. e., the scaled negative absorption spectrum.

Time correlated single photon counting and time resolved emission spectroscopy

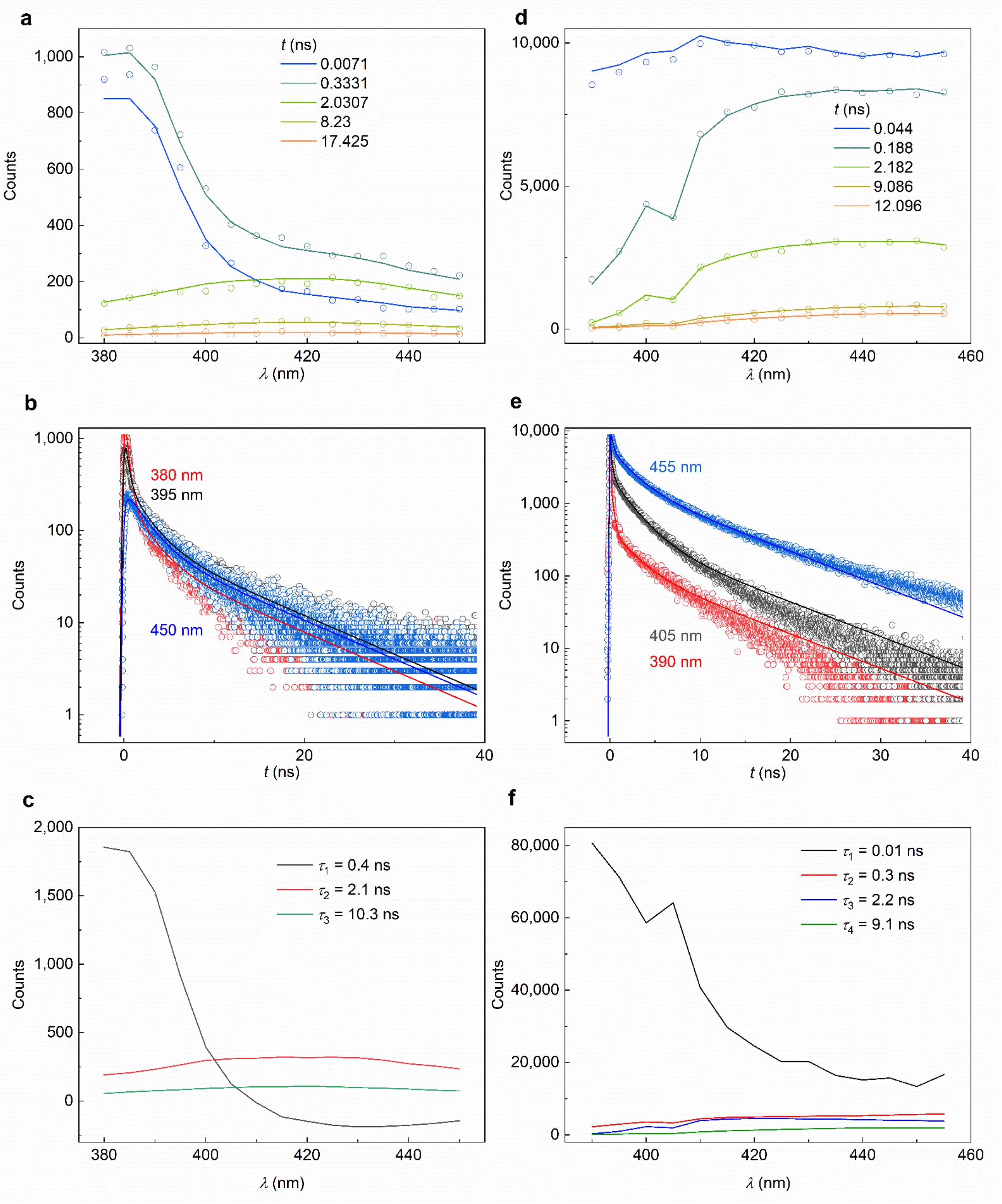

Supplementary Figure 19| TRES Data after excitation at 346 nm and 374 nm. a, b, c, measurements between 380 and 450 nm for 1 h per data point after 346 nm excitation. **a,** Decay of the fluorescence signal over time. **b,** Decay curves (points) at 380, 395 and 450 nm with the corresponding fits (line). **c,** Amplitude spectra of the lifetimes τ_1 = 0.4 ns, τ_2 = 2.1 ns and τ_3 = 10.3 ns obtained from global fitting. If the sub-ns lifetime is ignored, since it is attributed to stray-light of the excitation source, an averaged lifetime of 4.1 ns, comparable to the streak camera measurements is obtained. **d, e, f,** Measurements between 390 and 455 nm for 10k Counts per data point after 374 nm excitation. **d,** Decay of the fluorescence signal over time. **e,** Decay curves (points) at 390 nm, 405 nm and 455 nm with the respective fits (line). **f,** Amplitude spectra of the lifetimes τ_1 = 0.01 ns, τ_2 = 0.3 ns, τ_3 = 2.2 ns and τ_4 = 9.1 ns obtained from global fitting of the decay curves. If the sub-ns lifetimes are ignored here as well, an averaged lifetime of 4.0 ns is obtained.

Quenching experiments

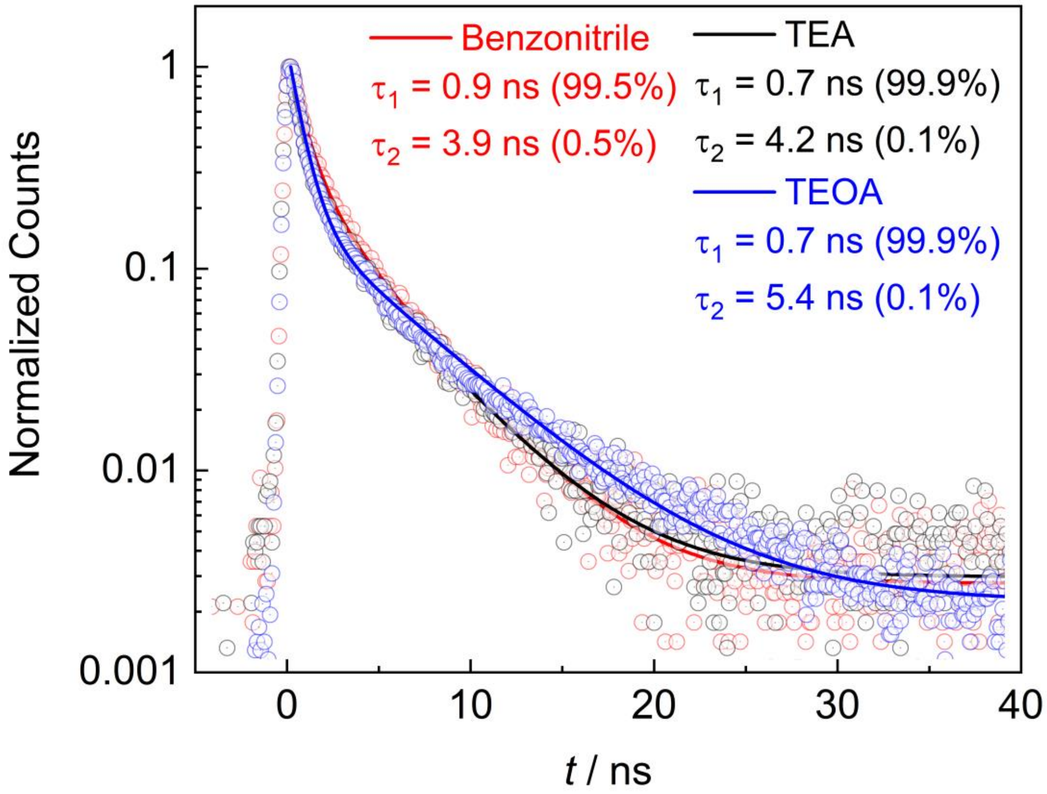

Supplementary Figure 20| Decay curves of the quenching experiments. The decay curves of the quenchers show a significant quenching of the excited state. In

all three quenching experiments, the short time constant dominates the decay, while the long time constant is almost completely quenched.

Singlet oxygen sensitization

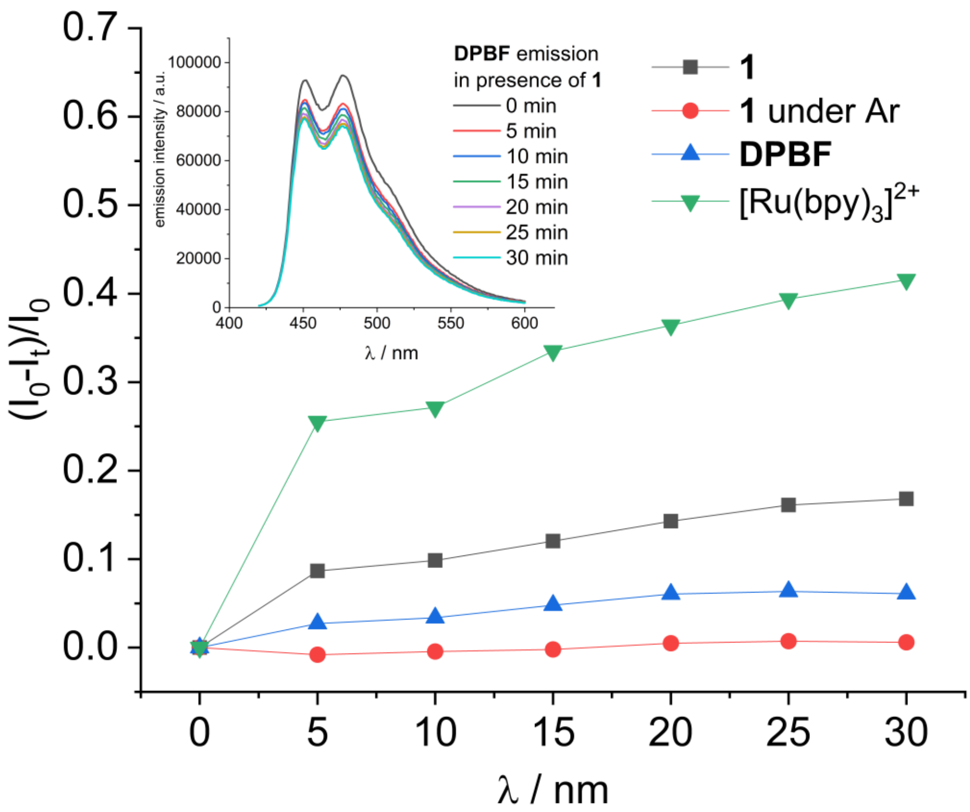

Supplementary Figure 21| DPBF consumption based on the fluorescence intensity as function of irradiation time (λ = 350 nm). Inset: Emission spectra of DPBF during irradiation.
