# Supplementary material for: Janus-type emission from a cyclometalated iron(iii) complex
Source: Nat Chem. 2023 Feb 27;15(4):468–74. doi: 10.1038/s41557-023-01137-w (PMC10070185; doi:10.1038/s41557-023-01137-w)
Supplement: Supplementary file 2 — DFT-calculated geometries as .xyz files and in a PDF. [file 41557_2023_1137_MOESM2_ESM.zip › DFT coordinates.pdf]

Coordinates from the DFT calculations in the respective charge and spin state.

$[Fe(ImP)_2]^+$, doublet state

Fe -0.013608 -0.003293 -0.018299

C -1.314946 2.508637 -0.729002

C -1.433346 3.883031 -0.933454

C -0.297455 4.673865 -0.693495

C 0.920455 4.122677 -0.261622

C -0.129181 1.920782 -0.299745

C 0.970580 2.742149 -0.072335

H -2.364980 4.344932 -1.268584

H -0.363257 5.752477 -0.847458

H 1.784146 4.767589 -0.083879

N -2.298097 1.501271 -0.912329

C -3.590295 1.515127 -1.415858

C -3.994112 0.215763 -1.442973

N -2.942660 -0.558422 -0.953161

C -1.876671 0.215813 -0.621280

N 2.063031 1.951392 0.371662

C 3.335205 2.260345 0.829420

C 3.889319 1.079185 1.216955

N 2.945877 0.079246 0.983474

C 1.801997 0.595722 0.463572

C 3.147216 -1.326643 1.314547

H 2.729466 -1.954521 0.520172

H 2.647709 -1.575363 2.261266

H 4.221745 -1.517849 1.410095

C -2.969012 -2.014165 -0.869862

H -2.494206 -2.337037 0.062965

H -2.425597 -2.458713 -1.715290

H -4.010891 -2.352446 -0.890355
H -4.104654 2.426971 -1.697312
H -4.933279 -0.228664 -1.753595
H 3.733619 3.268585 0.831385
H 4.868429 0.855228 1.625956
H -0.662356 1.509096 -3.465980
H -1.088824 2.484430 3.846747
C -0.582419 -1.406101 3.824613
C 0.762240 -1.214277 -3.999342
C 0.788387 -2.380598 -3.298474
C 0.420188 -0.665779 -1.822241
C -0.704184 -0.092275 4.158553
C 0.397709 1.219801 -3.456714
C -0.118913 -2.475474 1.527129
C -0.048127 -3.847548 1.764968
C 0.263686 -4.678730 0.676233
C 0.102274 -1.927155 0.267597
C 0.497007 -4.168532 -0.611577
C -0.624530 2.106115 2.929442
C -0.398993 -0.160382 1.909097
C 0.406642 -2.787501 -0.782771
N -0.594564 0.649030 2.982495
N -0.397200 -1.433478 2.450418
N 0.578600 -2.032117 -1.972398
N 0.541432 -0.183446 -3.086540
H 0.737287 -4.844088 -1.435717
H -0.620028 -2.302188 4.433696
H -0.869405 0.383377 5.119119
H 0.891781 -1.020456 -5.058430
H -1.206318 2.431085 2.060065
H 0.927971 1.847974 -2.732658

H 0.326742 -5.756624 0.836195

H 0.943465 -3.403127 -3.623623

H -0.223708 -4.277810 2.753610

H 0.393991 2.509607 2.843937

H 0.822724 1.368317 -4.455520

$[Fe(ImP)_2]^+$, quartet state

Fe -0.014935 -0.098238 -0.004861

C -1.316490 2.503490 -0.687979

C -1.427001 3.878446 -0.900934

C -0.290087 4.672014 -0.705441

C 0.925191 4.101803 -0.307485

C -0.129288 1.881556 -0.292009

C 0.973344 2.720775 -0.111542

H -2.368013 4.338353 -1.212638

H -0.351989 5.749778 -0.865540

H 1.803626 4.734466 -0.158394

N -2.394497 1.580076 -0.849208

C -3.717025 1.790153 -1.213827

C -4.309128 0.562592 -1.209028

N -3.329177 -0.354345 -0.841620

C -2.138761 0.252751 -0.614123

N 2.145320 2.011280 0.292634

C 3.425965 2.468033 0.570947

C 4.153761 1.366644 0.909445

N 3.293747 0.275901 0.826266

C 2.047661 0.651465 0.446799

C 3.671836 -1.103141 1.110356

H 2.788898 -1.733857 0.951878

H 4.009596 -1.199362 2.150329

H 4.477424 -1.423420 0.437142

C -3.540516 -1.791166 -0.715335

H -2.591182 -2.244803 -0.406291

H -3.854275 -2.216611 -1.677305

H -4.309228 -1.998452 0.040197

H -4.124002 2.769280 -1.440615

H -5.328831 0.265734 -1.431487

H 3.712679 3.511926 0.508140

H 5.194857 1.265505 1.198063

H 0.007226 1.187976 -4.348242

H -2.051608 2.177209 3.326984

C -0.811966 -1.624388 3.814241

C 1.126226 -1.394215 -3.980581

C 1.062041 -2.535837 -3.237728

C 0.531843 -0.769556 -1.880923

C -1.049100 -0.337170 4.196626

C 0.754261 1.058869 -3.554798

C -0.135181 -2.578277 1.530298

C -0.043850 -3.949757 1.765850

C 0.318757 -4.770450 0.686635

C 0.118000 -2.024755 0.279126

C 0.584593 -4.253703 -0.590813

C -1.017227 1.914429 3.071046

C -0.502087 -0.269581 1.994855

C 0.475428 -2.874142 -0.763274

N -0.854743 0.464801 3.077046

N -0.481244 -1.566838 2.472501

N 0.699302 -2.139419 -1.963380

N 0.801015 -0.337793 -3.136371

H 0.865445 -4.923470 -1.406623

H -0.851406 -2.550057 4.377741

H -1.336009 0.076004 5.157630

H 1.372110 -1.241429 -5.025890

H -0.781056 2.276964 2.064159

H 0.478135 1.663847 -2.683746

H 0.396994 -5.847270 0.846409

H 1.240846 -3.570149 -3.509995

H -0.243158 -4.388397 2.746022

H -0.335708 2.370957 3.799909

H 1.737749 1.371789 -3.927739

$[Fe(ImP)_2]^+$, sextet state

Fe 0.016576 0.099939 0.079584

C -1.362407 2.528201 -0.903928

C -1.442136 3.880767 -1.319378

C -0.308385 4.674152 -1.213392

C 0.932247 4.187822 -0.707882

C -0.141775 2.010652 -0.366907

C 0.963714 2.853309 -0.299878

H -2.368890 4.296342 -1.722236

H -0.365890 5.716065 -1.536509

H 1.805983 4.837660 -0.647122

N -2.323944 1.552849 -0.951028

C -3.660729 1.525148 -1.344453

C -4.100595 0.244986 -1.121764

N -3.064932 -0.498094 -0.612936

C -1.921901 0.282921 -0.490276

N 2.108659 2.164633 0.192187

C 3.406818 2.558437 0.459773

C 4.050794 1.442548 0.912666

N 3.125644 0.403451 0.904130

C 1.925522 0.836956 0.460383

C 3.405209 -0.961646 1.336602

H 2.535664 -1.583132 1.092704

H 3.584008 -0.985800 2.419036

H 4.288707 -1.344243 0.811676

C -3.172984 -1.886253 -0.201419

H -2.984569 -1.982782 0.877808

H -2.444071 -2.507725 -0.739377

H -4.185753 -2.241233 -0.422427

H -4.179280 2.391739 -1.737880

H -5.081774 -0.188759 -1.287009

H 3.760502 3.572538 0.311834

H 5.075346 1.295565 1.236136

H 0.381072 1.419547 -4.491673

H -1.712539 2.062807 3.958496

C -0.881013 -1.871264 3.819886

C 0.915688 -1.271377 -4.114356

C 0.751167 -2.403193 -3.375558

C 0.651337 -0.612625 -1.959628

C -1.023775 -0.642247 4.390665

C 1.011541 1.201963 -3.620738

C -0.217651 -2.546104 1.442686

C -0.241303 -3.933697 1.570703

C 0.017920 -4.703360 0.429369

C 0.050923 -1.908008 0.228519

C 0.306834 -4.111662 -0.805620

C -0.724776 1.742771 3.604015

C -0.369714 -0.298100 2.247781

C 0.323351 -2.718347 -0.877223

N -0.706747 0.296889 3.412709

N -0.479149 -1.639396 2.511057

N 0.593669 -1.980684 -2.061642

N 0.849183 -0.195231 -3.231788

H 0.514900 -4.735763 -1.677020

H -1.034452 -2.863207 4.229786

H -1.320822 -0.356313 5.394211

H 1.071803 -1.137398 -5.179621

H -0.512853 2.211744 2.635719

H 0.703714 1.830008 -2.775967

H 0.001033 -5.791749 0.506914

H 0.725082 -3.445242 -3.673959

H -0.453351 -4.421826 2.524099

H 0.038372 2.039905 4.334407

H 2.060282 1.408964 -3.871446

[Fe(ImP)$_2$], singlet state

Fe -0.004925 -0.000014 0.000492

C -1.339283 2.485701 -0.751922

C -1.461706 3.845180 -1.044047

C -0.320715 4.653291 -0.892339

C 0.906938 4.117495 -0.462458

C -0.143545 1.897574 -0.320114

C 0.959352 2.750436 -0.188806

H -2.404957 4.284577 -1.381840

H -0.389538 5.720950 -1.112339

H 1.781741 4.765613 -0.354739

N -2.324021 1.475323 -0.839461

C -3.669828 1.477481 -1.178585

C -4.088040 0.187164 -1.061239

N -2.991695 -0.572632 -0.656983

C -1.867030 0.199958 -0.506646

N 2.066777 1.980911 0.234990

C 3.384645 2.291682 0.537884

C 3.975186 1.118536 0.895270

N 3.008152 0.119099 0.801174

C 1.796641 0.619450 0.390028

C 3.259063 -1.275466 1.125085

H 2.355790 -1.846074 0.878993

H 3.483546 -1.390730 2.195530

H 4.108316 -1.656522 0.540751

C -3.043233 -2.003966 -0.398039

H -2.832797 -2.213070 0.659448

H -2.294535 -2.530567 -1.003213

H -4.045110 -2.371742 -0.649847

H -4.205725 2.374682 -1.468781

H -5.063098 -0.258520 -1.229121

H 3.783684 3.298463 0.474820

H 4.992278 0.903725 1.206809

H -0.110707 1.798214 -3.042165

H -1.816190 2.363479 3.318107

C -0.837189 -1.507277 3.750970

C 1.116733 -1.098833 -3.919603

C 1.008654 -2.292480 -3.273765

C 0.502305 -0.616370 -1.767846

C -1.001972 -0.203717 4.106394

C 0.843938 1.325960 -3.305286

C -0.207657 -2.516483 1.469387

C -0.204194 -3.898086 1.663713

C 0.108592 -4.714220 0.561350

C 0.084151 -1.912480 0.240168

C 0.408599 -4.164081 -0.697774

C -0.834044 2.022728 2.961315

C -0.452485 -0.204046 1.881838

C 0.388093 -2.773876 -0.821695

N -0.768131 0.570811 2.971354

N -0.503912 -1.498354 2.404167

N 0.637193 -1.989876 -1.971232

N 0.807063 -0.096957 -3.001410

H 0.646077 -4.819107 -1.541256

H -0.926748 -2.419401 4.331280

H -1.263197 0.242719 5.060420

H 1.383570 -0.871614 -4.946568

H -0.679195 2.359319 1.929442

H 1.637912 1.823652 -2.731873

H 0.119380 -5.799254 0.686283

H 1.161083 -3.307304 -3.624968

H -0.437122 -4.349204 2.632847

H -0.053145 2.450523 3.606773

H 1.035322 1.452444 -4.377616

$[Fe(ImP)_2]^{2+}$, triplet state

Fe -0.018295 0.017843 -0.001455

C -1.385002 2.500127 -0.607562

C -1.543684 3.879798 -0.720238

C -0.420067 4.682494 -0.441129

C 0.828370 4.145595 -0.071436

C -0.169092 1.921415 -0.228978

C 0.922608 2.758620 0.024293

H -2.490994 4.336800 -1.016291

H -0.519159 5.767298 -0.522886

H 1.678363 4.803654 0.123840

N -2.344792 1.479604 -0.841538

C -3.654824 1.485974 -1.283172

C -4.023395 0.171489 -1.369924

N -2.928042 -0.596762 -0.986844

C -1.881106 0.194011 -0.649014

N 2.054978 1.971433 0.363965

C 3.374358 2.267307 0.655181

C 3.992530 1.061502 0.842611

N 3.037443 0.067041 0.652656

C 1.830918 0.609360 0.363261

C 3.316035 -1.367001 0.723026

H 2.599339 -1.853779 1.398970

H 4.336791 -1.503579 1.101578

H 3.232742 -1.812215 -0.279759

C -2.911474 -2.059493 -0.989799

H -2.530345 -2.429648 -0.028197

H -2.266213 -2.424246 -1.803239

H -3.936770 -2.417729 -1.145797

H -4.204344 2.398348 -1.494522

H -4.963452 -0.284533 -1.666726

H 3.758099 3.281930 0.703634

H 5.021782 0.821674 1.093649

H -0.220686 1.592311 -3.180654

H -1.263106 2.553838 3.861927

C -0.897409 -1.322940 3.831672

C 1.143282 -1.269372 -3.838234

C 1.062945 -2.440276 -3.139651

C 0.530209 -0.721582 -1.736085

C -1.086595 0.001868 4.121639

C 0.788979 1.182945 -3.331446

C -0.214966 -2.430601 1.609965

C -0.171788 -3.794212 1.884864

C	0.190003	-4.659732	0.831334
C	0.080385	-1.909797	0.350414
C	0.497241	-4.186022	-0.451725
C	-0.936469	2.181186	2.882914
C	-0.513509	-0.112737	1.940393
C	0.432298	-2.804083	-0.660865
N	-0.847652	0.722383	2.958436
N	-0.548969	-1.367739	2.497927
N	0.686659	-2.086040	-1.852580
N	0.816072	-0.231952	-2.966255
H	0.773761	-4.879521	-1.249573
H	-0.980659	-2.210020	4.452528
H	-1.369556	0.494075	5.047499
H	1.403479	-1.077491	-4.875140
H	-1.665048	2.477309	2.115624
H	1.506469	1.744217	-2.715007
H	0.231309	-5.734119	1.023576
H	1.238754	-3.467800	-3.442533
H	-0.407155	-4.193751	2.874387
H	0.047116	2.606999	2.639300
H	1.066410	1.273141	-4.389304
